# Neuro-Geometric Graph Transformers with Differentiable Radiographic Geometry for Spinal X-Ray Image Analysis

**DOI:** 10.3390/jimaging12020059

**Published:** 2026-01-28

**Authors:** Vuth Kaveevorayan, Rapeepan Pitakaso, Thanatkij Srichok, Natthapong Nanthasamroeng, Chutchai Kaewta, Peerawat Luesak

**Affiliations:** 1 Orthopedic Department, Sunpasittiprasong Hospital, Ubon Ratchathani 34000, Thailand; vuthkav@yahoo.com; 2 Artificial Intelligence Optimization SMART Laboratory, Industrial Engineering Department, Faculty of Engineering, Ubon Ratchathani University, Ubon Ratchathani 34190, Thailand; rapeepan.p@ubu.ac.th; 3 Artificial Intelligence Optimization SMART Laboratory, Engineering Technology Department, Faculty of Industrial Technology, Ubon Ratchathani Rajabhat University, Ubon Ratchathani 34000, Thailand; natthapong.n@ubru.ac.th; 4 Digital Innovation, Faculty of Computer Science, Ubon Ratchathani Rajabhat University, Ubon Ratchathani 34000, Thailand; chutchai.k@ubru.ac.th; 5 Department of Industrial Engineering, Faculty of Engineering, Rajamangala University of Technology Lanna, Chiang Rai 57120, Thailand; peerawat@rmutl.ac.th

**Keywords:** radiographic imaging, neuro-geometric deep learning, graph transformers, differentiable radiographic indices, explainable medical image analysis

## Abstract

Radiographic imaging remains a cornerstone of diagnostic practice. However, accurate interpretation faces challenges from subtle visual signatures, anatomical variability, and inter-observer inconsistency. Conventional deep learning approaches, such as convolutional neural networks and vision transformers, deliver strong predictive performance but often lack anatomical grounding and interpretability, limiting their trustworthiness in imaging applications. To address these challenges, we present SpineNeuroSym, a neuro-geometric imaging framework that unifies geometry-aware learning and symbolic reasoning for explainable medical image analysis. The framework integrates weakly supervised keypoint and region-of-interest discovery, a dual-stream graph–transformer backbone, and a Differentiable Radiographic Geometry Module (dRGM) that computes clinically relevant indices (e.g., slip ratio, disc asymmetry, sacroiliac spacing, and curvature measures). A Neuro-Symbolic Constraint Layer (NSCL) enforces monotonic logic in image-derived predictions, while a Counterfactual Geometry Diffusion (CGD) module generates rare imaging phenotypes and provides diagnostic auditing through counterfactual validation. Evaluated on a comprehensive dataset of 1613 spinal radiographs from Sunpasitthiprasong Hospital encompassing six diagnostic categories—spondylolisthesis (*n* = 496), infection (*n* = 322), spondyloarthropathy (*n* = 275), normal cervical (*n* = 192), normal thoracic (*n* = 70), and normal lumbar spine (*n* = 258)—SpineNeuroSym achieved 89.4% classification accuracy, a macro-F1 of 0.872, and an AUROC of 0.941, outperforming eight state-of-the-art imaging baselines. These results highlight how integrating neuro-geometric modeling, symbolic constraints, and counterfactual validation advances explainable, trustworthy, and reproducible medical imaging AI, establishing a pathway toward transparent image analysis systems.

## 1. Introduction

Radiographic imaging remains a primary tool in the assessment of spinal anatomy and pathology, offering broad accessibility, low cost, and reduced radiation exposure compared to modalities such as computed tomography (CT) and magnetic resonance imaging (MRI). Despite its central role, spinal radiograph interpretation presents significant challenges. Subtle features such as early disc-space narrowing, vertebral endplate irregularities, and minor displacements complicate analysis. These features are often difficult to distinguish from normal anatomical variations. These challenges contribute to variability between observers and limit diagnostic reliability, particularly in settings where access to specialized musculoskeletal radiology expertise is limited. Advances in imaging analysis and artificial intelligence provide new opportunities to address these challenges by enhancing reproducibility, improving accuracy, and introducing interpretable computational methods into radiographic assessment.

Deep learning (DL) methodologies have achieved transformative success in medical imaging applications, ranging from chest radiography and mammography to musculoskeletal ultrasound and spine imaging. For example, Bhuvanya et al. [1] proposed a hybrid ViT–Xception model for chest radiograph classification, Marzola et al. [2] introduced CNN-based segmentation for musculoskeletal ultrasound, and He et al. [3] developed a multimodal network for spine MRI segmentation. Despite these advances, key limitations persist. Predominantly, existing models lack clinical interpretability, operate as opaque black boxes, and fail to provide radiographic indices familiar to clinicians. Additionally, many models do not incorporate anatomical constraints, which can result in plausible-looking but clinically inconsistent outputs, as shown in studies by Yang et al. [4] and Duan et al. [5]. Rare and underrepresented phenotypes remain particularly vulnerable to misclassification due to data imbalance, a challenge emphasized by Ngasa et al. [6].

Emerging research has explored a variety of solutions to address these shortcomings. Ahmed et al. [7] introduced FedGraphMRI-net, a federated learning architecture that preserves spatial consistency across institutions. Zheng et al. [8] employed graph-based networks to retain anatomical structure, while Belmonte et al. [9] incorporated symbolic logic into hybrid models for brain tissue segmentation. To enhance robustness to rare cases, Mulé et al. [10] utilized GANs for rare liver cancer synthesis, and Roschewitz et al. [11] proposed counterfactual contrastive learning for domain-shift resilience. Weakly supervised learning and LLM-augmented training pipelines have also been developed to reduce annotation burden and improve classification [12,13]. However, these approaches often lack the integration of explicit geometric reasoning crucial for spinal interpretation.

Despite these innovations, critical research gaps remain. For instance, while He et al. [3] focused on multi-modal spine MRI segmentation, and Verma et al. [14] explored spinal cord injury biomarkers with MRI and DTI, neither addressed the interpretability of radiographic indices. Cronin et al. [15] automated fascicle segmentation in ultrasound, but without symbolic constraint logic. Gao et al. [16] addressed continual learning in sonographic data, yet geometric measurements were not considered. Ngasa et al. [6] and Roschewitz et al. [11] demonstrated generative and counterfactual reasoning, but applied these to domains outside spinal radiography and without incorporation of clinically relevant metrics such as slip ratio or Cobb angle.

Recent advances in explainable artificial intelligence (XAI) and biomarker discovery have fundamentally transformed medical image analysis paradigms, particularly in musculoskeletal radiology. The dichotomy between handcrafted radiological features and deep learned representations has emerged as a critical consideration for clinical translation, with evidence suggesting that hybrid approaches combining domain expertise with data-driven learning yield superior interpretability and diagnostic reliability [17]. Traditional radiological biomarkers—such as vertebral slip ratios, disc space measurements, and spinal alignment angles—provide clinicians with quantitative, interpretable metrics that align with established diagnostic protocols. However, conventional deep learning approaches often bypass these established biomarkers in favor of abstract feature representations, creating a disconnect between AI predictions and radiological reasoning.

Contemporary XAI frameworks emphasize the importance of evidence-based design principles that integrate clinical domain knowledge into algorithmic decision-making processes. As demonstrated by [18], effective decision support systems must transcend post hoc explanations to embed interpretability mechanisms directly into model architecture, ensuring that AI outputs align with clinical reasoning pathways. In spinal imaging, this translates to systems that generate not only diagnostic classifications but also quantitative measurements familiar to radiologists—slip ratios for spondylolisthesis grading, disc asymmetry indices for infection detection, and curvature angles for alignment assessment. Such approaches bridge the interpretability gap while maintaining predictive performance, addressing the fundamental challenge of clinical AI adoption in radiology departments where transparency and accountability are paramount.

To address these gaps, we propose SpineNeuroSym, a novel, geometry-aware, neuro-symbolic, and counterfactual generative framework for spinal radiograph interpretation. The system incorporates: (i) a weakly supervised key point–ROI discovery module for anatomy localization; (ii) a dual-stream graph-transformer backbone for local–global reasoning; (iii) a differentiable radiographic geometry module (dRGM) that computes clinically interpretable indices; (iv) a neuro-symbolic constraint layer (NSCL) that encodes domain logic as differentiable constraints; and (v) a counterfactual geometry diffusion (CGD) module that generates rare phenotypes and validates monotonicity. Collectively, these innovations enable interpretable, trustworthy, and clinically aligned spinal diagnosis.

The principal contributions of this study are:

1. A unified diagnostic pipeline that integrates weak supervision, geometric reasoning, neuro-symbolic constraints, and counterfactual augmentation.
2. Differentiable outputs that map directly to radiographic indices used in clinical practice, enhancing interpretability.
3. Robust generalization to rare or underrepresented spinal phenotypes through generative counterfactual modeling.
4. Demonstrated clinical readiness via benchmarking against current baselines, radiologist agreement studies, and deployment in a prototype decision support tool.

The remainder of this manuscript is organized as follows. Section 2 provides a comprehensive review of related work in deep learning foundations, vision transformers, graph neural networks, interpretability frameworks, neuro-symbolic integration, and generative models for medical imaging. Section 3 details our methodology, including dataset preparation, weakly supervised anatomy discovery, spine graph construction with dual-stream transformers, differentiable radiographic geometry computation, neuro-symbolic constraint integration, and counterfactual geometry diffusion. Section 4 presents comprehensive experimental results covering diagnostic performance, geometric plausibility validation, symbolic constraint effectiveness, counterfactual generation quality, and ablation studies. Section 5 provides detailed discussion of our findings within the context of existing literature, clinical implications, deployment considerations, and study limitations. Finally, Section 6 concludes with a summary of contributions and directions for future research in clinically grounded medical AI systems.

## 2. Related Work

### 2.1. Deep Learning Foundations in Spinal Radiography

Convolutional neural networks (CNNs) have established the methodological foundation for spinal imaging applications, encompassing vertebral fracture detection, stenosis identification, and anatomical segmentation. Contemporary developments demonstrate significant clinical utility across diverse diagnostic challenges.

CNN-based architectures have achieved radiologist-level performance in lumbar spinal canal stenosis detection [19], while automated pelvic parameter measurement frameworks have enhanced pre-surgical planning accuracy in complex spinal deformities [20]. Advanced spatial configuration networks incorporating attention mechanisms have further improved vertebral fracture detection and level assessment through multilabel segmentation approaches, significantly reducing diagnostic redundancy [21].

Clinical workflow integration has been enhanced through systematic CT-based vertebral fracture reporting pipelines [22] and dual-energy CT reconstruction algorithms that improve compression fracture evaluation precision [23]. Novel architectural developments include DiffCNN, which combines CNNs with diffusion models for semi-supervised segmentation, demonstrating reduced data dependency while maintaining accuracy [24].

Expanding beyond traditional imaging modalities, graph convolutional networks have shown promise for spinal cord injury detection through EEG decoding, representing interdisciplinary convergence between neuroimaging and spinal diagnostics [25]. These advances collectively indicate progression toward more accurate, interpretable, and clinically integrated AI solutions.

### 2.2. Vision Transformers and Global Context Integration

Vision Transformers (ViTs) represent a paradigmatic advancement in medical imaging through global context modeling via self-attention mechanisms, transcending the spatial locality constraints inherent in CNN architectures. This capability enables holistic radiograph analysis, particularly advantageous for multi-regional spinal pathology assessment across cervical, thoracic, and lumbar segments [26].

Multi-axis transformer architectures demonstrate superior segmentation accuracy in complex spatial variance scenarios compared to conventional CNNs [26]. Hybrid CNN-transformer models have shown enhanced performance in parallel medical applications, suggesting translational potential for spinal imaging contexts [27].

However, interpretability limitations persist as a critical barrier to clinical adoption. Integration of Local Interpretable Model-Agnostic Explanations (LIME) with ViT architectures addresses this challenge by providing decision boundary visualization aligned with clinical understanding [28].

Clinical translation necessitates addressing broader considerations of equity, transparency, and contextual applicability. Expert consensus emphasizes that successful AI integration requires interpretable, transparent architectures that generalize across diverse populations and resource settings [28,29,30].

While ViTs demonstrate compelling capabilities in capturing global dependencies and enhancing diagnostic performance, clinical integration depends critically on developing architectures that reconcile computational power with explainability, aligned with radiological practice standards and ethical frameworks.

### 2.3. Graph Neural Networks for Anatomical Representation

Graph Neural Networks (GNNs) address fundamental limitations of convolution-based architectures by modeling anatomical structures as interconnected networks, where vertebrae and intervertebral discs represent nodes and their biomechanical relationships constitute edges. This paradigm enables unified learning of local pathologies and global spinal alignment patterns.

GNN frameworks demonstrate superior performance in recognizing structured spatial dependencies compared to conventional CNNs. Node-level capsule GNNs achieve enhanced fracture discrimination on radiographs [31], while multi-view GNN models improve MRI-based joint injury classification through cross-planar spatial integration [32]. Anatomically aware modeling extends to musculature quantification, with automated tools demonstrating clinical utility in subject-specific spinal deformity modeling and surgical planning [33].

Methodological advances include IncARMAG, which integrates GNNs with autoregressive moving average operations to capture multi-level anatomical correlations [34]. However, data-driven GNNs lack biological plausibility constraints, potentially generating anatomically unrealistic predictions.

Biologically plausible learning paradigms address these limitations through local derivative approximation with memory replay [35] and spatiotemporal error propagation mechanisms [36]. Integration with predictive frameworks for spinal reconstruction, including hydrogel-based therapies evaluated through image-guided models, demonstrates expanding clinical applications [37].

GNNs show exceptional promise for anatomical reasoning in spinal imaging, contingent upon incorporating domain-specific biological constraints, interpretability mechanisms, and multimodal data integration for clinical translation.

### 2.4. Interpretability and Explainable AI in Medical Imaging

Interpretability has emerged as a critical prerequisite for clinical AI deployment, with radiologists demanding transparent systems aligned with anatomical observations and radiographic measurements [38]. Black-box models generating opaque outputs present diagnostic safety concerns, particularly when predictions contradict established clinical patterns.

Traditional interpretability approaches utilizing saliency maps, Grad-CAM, SHAP, and LIME provide visual attention proxies but lack causal alignment with model decisions and structured radiological reasoning [39]. Advanced approaches like DC-Net integrate saliency decomposition directly into segmentation pipelines, maintaining spatial fidelity while enhancing interpretability [40].

Multimodal frameworks demonstrate enhanced clinical relevance through structured feature integration. Zheng et al. [8] developed edge detection and salient feature extraction models for MRI-CT fusion, revealing clinically relevant structural insights. Similarly, patch-based interpretable architectures for multimodal neuroimaging demonstrate improved clinical acceptance through spatially constrained feature representations [41].

Systematic evaluations using standardized explainability frameworks reveal significant shortcomings in current AI interpretability reporting. Comprehensive reviews of musculoskeletal tumor imaging tools identify major gaps in explainability documentation, emphasizing the need for regulatory standards [42].

Recent developments extend interpretability beyond visual explanations. Transformer-based models like WCFormer demonstrate interpretable temporal embeddings applicable across medical domains [43], suggesting translational potential for imaging applications.

Clinical-grade explainability requires progression beyond post hoc attention mechanisms toward intrinsically interpretable architectures incorporating multimodal integration and domain-specific metrics. Developing models that provide clinically rational justifications alongside predictions remains essential for safe, ethical AI deployment in medical imaging.

### 2.5. Neuro-Symbolic Integration in Clinical AI

Neuro-symbolic AI represents a paradigmatic convergence of symbolic reasoning and neural computation, addressing fundamental limitations in domain knowledge encoding and anatomical constraint adherence in healthcare applications. Traditional deep learning models frequently generate predictions violating clinical logic, while neuro-symbolic architectures embed structured knowledge—including logic rules, ontologies, and clinical guidelines—directly into neural frameworks, enabling explainable, constraint-aware learning [44].

Medical imaging applications demonstrate promising outcomes through symbolic integration. CNN frameworks incorporating clinical rules enhance anatomical plausibility in dental imaging, improving tooth detection accuracy [45]. Hybrid models like NeSyKHG (Neuro-Symbolic Knowledge Hypergraphs) provide structured hierarchical biomedical relationship representation, enabling robust inference in complex diagnostic pipelines [46].

Knowledge graphs and symbolic logic integration show significant potential for healthcare decision support. Prolog-based symbolic engines combined with large language models demonstrate improvements in domain-specific question answering and knowledge-grounded AI applications [47], facilitating statistical, semantic, and deductive reasoning capabilities essential for regulatory acceptance and clinical adoption.

However, symbolic integration in spinal imaging remains nascent, with implementations primarily addressing conceptual domains rather than direct radiographic index alignment. While neuro-symbolic approaches show potential in 4D-printing design contexts [48], translation to measurable spinal imaging outputs requires novel architectures incorporating anatomical ontologies, clinical guidelines, and symbolic abstraction principles observed in cognitive systems [49,50,51].

Neuro-symbolic integration offers significant potential for transparent, knowledge-aligned clinical AI, yet application to spinal imaging and quantitative radiographic assessment remains an open research frontier requiring architecture guided by domain rules and interpretability mandates.

### 2.6. Generative Models for Data Augmentation and Rare Phenotypes

Generative Adversarial Networks (GANs) and diffusion-based architectures have transformed medical imaging data augmentation by addressing labeled data scarcity, particularly for rare pathologies, through realistic radiographic synthesis that expands training corpora and improves cross-domain generalization.

Advanced augmentation methodologies demonstrate enhanced robustness in segmentation and classification tasks. Feature style augmentation strategies modulate texture diversity while preserving semantic structure, improving performance under domain shift conditions [52]. Cross-domain attention-guided augmentation pipelines generate clinically plausible samples, particularly effective with limited training data [53].

Despite visual realism, concerns persist regarding clinical relevance and diagnostic validity of generated data. Multi-Stage Gaussian Diffusion Generation (MSDGD) frameworks address these limitations by incorporating statistical controls aligning outputs with diagnostic features [54]. Volumetric GAN applications enable 3D synthesis critical for spatially complex pathologies, with systematic reviews highlighting anatomically coherent data generation across CT and MRI modalities [55].

Multimodal generative applications extend beyond image synthesis to textual augmentation. Radiology report generation pipelines combining automatic keyword adaptation with large language models enhance narrative labeling capabilities [56], while vision-language captioning frameworks facilitate AI-human collaboration in clinical reporting [57].

Principled approaches addressing semantic drift and causality preservation incorporate clinical metadata insights, ensuring synthetic data alignment with real-world disease progression and comorbidity patterns [58]. Future development priorities include enhancing clinical validity, anatomical accuracy, and diagnostic relevance evaluation beyond visual plausibility assessment.

### 2.7. Research Gaps and Study Rationale

Contemporary deep learning approaches in spinal imaging exhibit significant methodological limitations despite notable advances. CNN-based models demonstrate effectiveness in spinal stenosis detection, pelvic parameter measurement, and vertebral fracture identification [19,20,21], yet remain constrained by local receptive fields, lack anatomical grounding, and produce opaque predictions without clinically familiar radiographic indices.

Vision Transformer architectures improve global contextual reasoning and outperform CNNs in large-scale imaging tasks [26,27,28], but continue functioning as opaque classifiers with interpretability achieved only through post hoc visualizations rather than embedded mechanisms.

Graph Neural Networks capture anatomical relationships more explicitly through vertebral and musculoskeletal structure representation as interconnected nodes and edges [31,32,33], yet remain purely data-driven, often producing biologically implausible outputs without symbolic or clinical constraints.

Interpretability research emphasizes saliency maps, patch-based models, and formal explainability frameworks [39,40,41,42], but these methods remain external to training processes, offering descriptive rather than intrinsic interpretability, limiting clinical reasoning pathway integration.

Neuro-symbolic AI demonstrates potential for embedding symbolic rules into neural networks, enforcing anatomical plausibility and semantic reasoning [44,45,46], yet frameworks lack direct spinal radiograph application or integration of measurable indices such as slip ratio or Cobb angle.

Generative modeling approaches confirm GANs and diffusion models can synthesize realistic radiographs and improve domain shift robustness [52,53,55], but prioritize pixel-level fidelity over diagnostic validity without validating diagnostic consistency under targeted geometric alterations.

These limitations indicate an unresolved need for unified frameworks embedding anatomical reasoning, enforcing symbolic constraints, and leveraging counterfactual validation. Existing approaches lack intrinsic interpretability, clinical prior integration, diagnostic consistency validation, and spinal-specific neuro-symbolic application. This study addresses these shortcomings through SpineNeuroSym, integrating geometry-aware modeling, neuro-symbolic constraints, and counterfactual diffusion within a clinically aligned decision-support framework.

## 3. Methods

**Figure 1:**
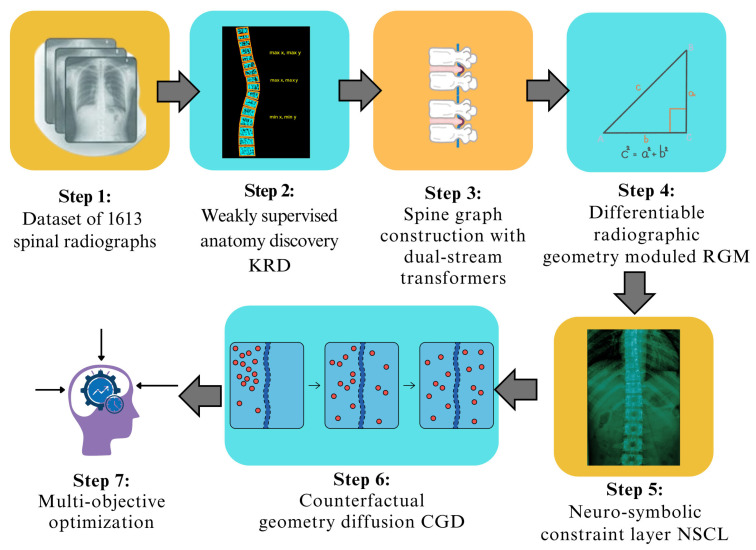
Stepwise workflow of the proposed methodology.

The overall SpineNeuroSym framework is summarized in Figure 1, which illustrates the sequential workflow of the proposed methodology. Our methodology processes 1613 spinal radiographs through six main stages. First, the KRD module localizes vertebral centroids and disc regions using weakly supervised learning. Next, we organize these landmarks into graph structures using dual-stream transformers for local and global feature analysis. The dRGM module then computes clinically relevant measurements including slip ratio, disc asymmetry, and spinal curvature. These indices are formulated as differentiable functions that integrate seamlessly with neural network training. Our NSCL component enforces clinical reasoning by embedding radiological knowledge as symbolic constraints. The CGD module generates synthetic radiographs to improve robustness for rare pathological presentations. Finally, we optimize the complete framework using a multi-objective loss function that balances diagnostic accuracy with geometric plausibility and clinical consistency. Figure 1 shows the stepwise workflow of the proposed methodology that we have been discussed.

### 3.1. Dataset

We retrospectively collected 1613 anonymized spinal X-ray images from Sunpasitthiprasong Hospital in Ubon Ratchathani, Thailand. This institution serves as a major tertiary care and referral center for northeastern Thailand. All imaging data were acquired using standard digital radiography protocols and stored in DICOM format before being anonymized. Ethical approval for data usage was obtained from the hospital’s institutional review board, and no personally identifiable information or clinical metadata (e.g., age, sex, comorbidities) was included, thereby ensuring patient privacy and focusing the study exclusively on image-based diagnostic performance.

Labeling protocol. Each X-ray image was independently reviewed and annotated by two board-certified radiologists with over 15 years of experience in musculoskeletal imaging. The labeling process followed a dual-review consensus strategy: in cases of initial disagreement, the radiologists conducted a consensus discussion to determine the final label. This process ensured high diagnostic reliability and consistency across the dataset. Based on clinically established diagnostic criteria, each X-ray image was assigned to one of six diagnostic categories relevant to spinal pathology and anatomical normalcy:

1. Spondylolisthesis (*n* = 496, 30.7%)—cases exhibiting vertebral slippage or anterior/posterior displacement indicative of structural instability.
2. Infection (*n* = 322, 20.0%)—images showing evidence of spondylodiscitis, endplate erosion, disc space narrowing, or other radiologic signs of spinal infection.
3. Spondyloarthropathy (SpA) (*n* = 275, 17.0%)—including inflammatory spinal disorders such as ankylosing spondylitis, with hallmark features such as vertebral squaring, syndesmophytes, and sacroiliac joint erosion.
4. Normal cervical spine (*n* = 192, 11.9%)—X-ray images of the cervical spine with no radiographic abnormalities.
5. Normal thoracic spine (*n* = 70, 4.3%)—thoracic spine images demonstrating normal vertebral alignment and structure.
6. Normal lumbar spine (*n* = 258, 16.0%)—lumbar spine images free of pathological findings.

This categorization was designed to balance representation across both pathological and non-pathological cases, while capturing anatomical diversity across the cervical, thoracic, and lumbar spine regions. Such stratification is essential to evaluate the classification model’s ability to distinguish between visually similar conditions and to generalize across spinal levels.

Dataset distribution. The overall distribution of X-ray images across diagnostic categories is presented in Table 1. While the dataset is moderately imbalanced, with spondylolisthesis being the most prevalent condition, it nonetheless includes substantial representation from each diagnostic class. This imbalance was addressed during model training through class-balanced sampling techniques and targeted data augmentation strategies.

**Figure 2:**
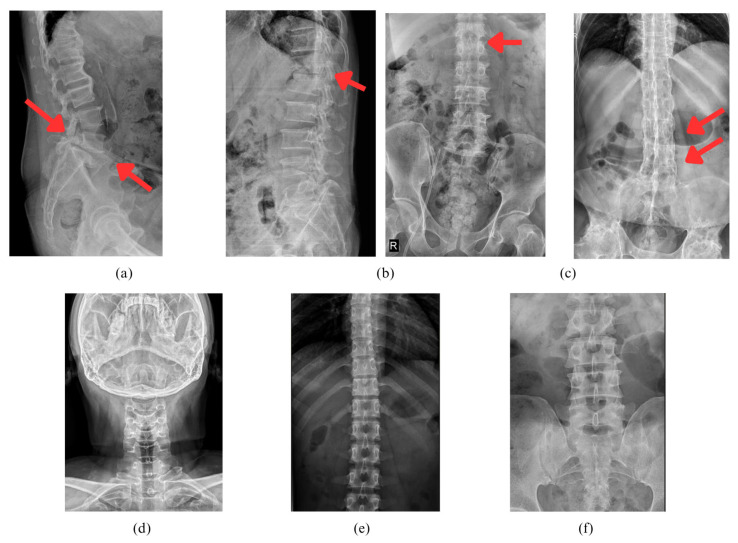
Representative spinal X-ray images for each diagnostic class: (**a**) spondylolisthesis, (**b**) infection, (**c**) spondyloarthropathy (SpA), (**d**) normal cervical spine, (**e**) normal thoracic spine, and (**f**) normal lumbar spine.

Representative examples of each diagnostic class are provided in Figure 2, which visually demonstrates the heterogeneity of spinal X-ray appearances. These examples contextualize the complexity of automated image classification by highlighting how disease-related radiographic patterns—such as endplate irregularities in SpA or subtle infection-related disc changes—may resemble normal anatomical variations. Accurate classification requires models to discern these nuanced features under conditions of intra-class variability and inter-class similarity.

In summary, this dataset forms a robust and clinically realistic foundation for developing and evaluating automated diagnostic models for spinal pathology. The inclusion of both pathological and normal cases across the full range of spinal regions ensures that the classification task reflects diagnostic scenarios commonly encountered in real-world radiological workflows.

### 3.2. Weakly Supervised Anatomy Discovery

To localize spinal anatomical structures without the burden of dense annotation, we developed a Weakly Supervised Keypoint–ROI Discovery (KRD) module that learns to identify vertebral centroids and intervertebral disc regions from a limited subset of annotated images. This module serves as the first step in constructing graph-based spinal representations for downstream analysis (detailed in Section 3.3).

Training with sparse supervision. The KRD model was trained on a sparsely labeled dataset in which only ≤15% of the total X-ray images contained manually annotated vertebral landmarks. These annotations were provided by musculoskeletal radiologists and included the center coordinates of visible vertebral bodies and adjacent disc spaces. This limited supervision regime significantly reduces labeling overhead while preserving anatomical fidelity.

Multi-source learning signals. To enhance generalization beyond the small labeled set, the KRD training strategy integrated three complementary learning signals:

1. Sparse expert supervision: Direct regression of annotated key point locations was applied where ground-truth labels were available.
2. Geometric self-supervision: In unlabeled images, we imposed a set of geometry-based priors that encode biological plausibility. These included:
  ○ Sequential ordering of vertebrae along the spine axis,
  ○ Smooth spacing constraints between adjacent centroids,
  ○ Left–right symmetry with respect to the spinal axis, and
  ○ Natural spinal curvature continuity (e.g., lordosis and kyphosis).

These priors were implemented via differentiable penalty terms in the loss function, enabling the network to infer plausible vertebral locations even in the absence of annotations.

3. Cross-view consistency via teacher–student distillation: To improve robustness to variation in posture, occlusion, and noise, we employed a teacher–student architecture. The teacher network—updated using an exponential moving average of the student weights—produced stable pseudo-labels across multiple geometric and photometric augmentations. The student was then trained to match the teacher’s heatmaps, thereby enforcing consistency across views and improving spatial confidence in ambiguous regions.

Output and anatomical representations. For each input X-ray image, the trained KRD model outputs:

- A set of vertebral key points representing predicted centroid locations along the spinal axis,
- Corresponding intervertebral disc region proposals as rectangular bounding boxes (ROIs),
- And a soft visibility heatmap, indicating the spatial certainty of key point detections.

These outputs form the basis for subsequent graph-based modeling by defining the node positions and receptive fields used in the spinal graph construction.

In summary, the weakly supervised anatomy discovery framework enables scalable, anatomically aware localization of spinal landmarks from large-scale X-ray datasets, with minimal dependence on manual annotation. This approach reflects a practical trade-off between clinical realism and model scalability, consistent with prior work on semi-supervised medical structure discovery.

**Figure 3:**
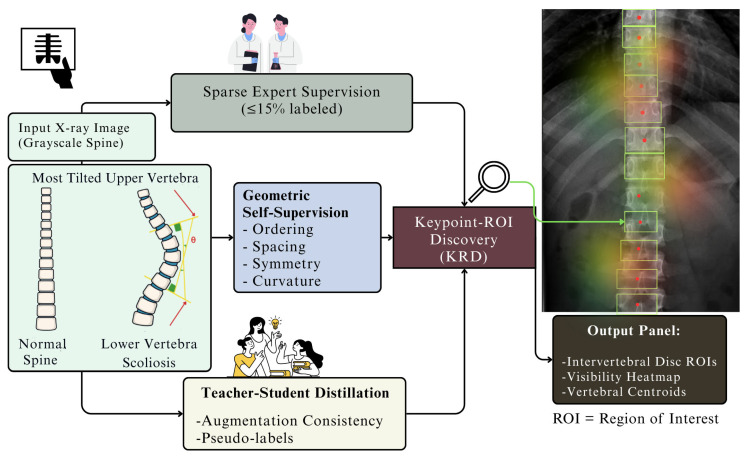
Overview of the weakly supervised key point–ROI discovery (KRD) pipeline.

Figure 3 illustrates the architecture of the proposed Weakly Supervised Keypoint–ROI Discovery (KRD) pipeline. The model integrates sparse expert annotations, geometric self-supervision, and teacher–student distillation to accurately infer vertebral landmarks and intervertebral disc regions from spinal X-ray images. This modular design enables robust anatomical localization with minimal manual labeling, forming the basis for subsequent graph-based spinal modeling.

### 3.3. Spine Graph Construction and Dual-Stream Transformer

To effectively model the spatial and contextual dependencies within spinal anatomy, we construct a graph-based representation of each X-ray image and process it using a dual-stream Transformer architecture. This design allows the model to reason jointly over local region-of-interest (ROI) features and global anatomical context, thereby improving diagnostic sensitivity for complex spinal disorders.

Graph construction from anatomical key points

Each input spinal X-ray is first converted into a structured graph $G=\left(V,E\right)$, where

- $V={v_{i}}_{i=1}^{N}$ denotes the set of nodes, corresponding to predicted vertebral centroids and intervertebral disc ROIs extracted by the KRD module (see Section 3.2),
- $E=e_{ij}$ represents the set of undirected edges, constructed based on spatial adjacency between anatomical landmarks.

Each edge $e_{ij}$ is enriched with a vector of geometric attributes (Equation (1)):

(1) $$a_{ij}=\left[∥\;p_{i}-p_{j}{∥}_{2},\;\theta_{ij},\;\kappa_{ij}\right]$$

where $∥p_{i}-p_{j}{∥}_{2}$ is the Euclidean distance between keypoints, $\theta_{ij}$ is the relative orientation angle, and $\kappa_{ij}$ encodes local curvature approximated using second-order differences along the spine axis. This anatomically informed representation preserves both the topological and geometric structure of the vertebral chain.

Dual-Stream Feature Encoding

To capture both fine-grained local features and holistic spatial dependencies, we employ a dual-stream architecture comprising:

- A local feature stream using a ConvNeXt encoder to extract discriminative representations from cropped ROI patches around each predicted node $v_{i}$, capturing local textural and morphological patterns (e.g., vertebral compression, disc narrowing).
- A global feature stream using a Swin Vision Transformer (Swin-ViT) applied to the entire X-ray image, which models long-range dependencies and global anatomical alignment.

The final node embedding $h_{i\;}$ for each anatomical site is constructed by concatenating features from both streams (Equation (2)):

(2) $$h_{i\;}=[\;h_{i}^{local}∥h_{i}^{global}\;\;]$$

where $h_{i}^{local}\in R^{d_{l}}$ and $h_{i}^{global}\in R^{d_{g}}$ are the outputs of the local and global encoders, respectively.

Graph Transformer for Joint Reasoning

The fused graph is then passed to a Graph Transformer module, which performs edge-aware attention across nodes to capture complex spatial dependencies and anatomical interactions. The attention score between nodes $v_{i}$ and $v_{j}$ is defined as (Equation (3)):

(3) $$Attention\left(q_{i},k_{j},a_{ij}\right)=\frac{{\left(q_{i}+\phi \left(a_{ij}\right)\right)}^{⊤}k_{j}}{d_{k}}$$

where $q_{i}$ and $k_{j}$ are query and key vectors are derived from node embeddings, and $\phi \left(a_{ij}\right)$ is a learnable transformation of the edge attributes $a_{ij}$, allowing anatomical priors to modulate attention weights.

The attention mechanism is followed by multi-layer message passing and feedforward updates (Equation (4)):

(4) $$h_{i}^{\prime }=MLP(\sum_{j\in N\left(i\right)}Attention\left(q_{i},k_{j},a_{ij}\right)\cdot v_{j})$$

This enables the model to integrate localized pathological signals (e.g., vertebral erosion) with contextual cues (e.g., multi-level degeneration or curvature anomalies), which are often critical for clinical diagnosis.

This module transforms each spinal X-ray into a richly structured graph and jointly processes it using anatomical feature fusion and graph-based reasoning. By combining node-level focus with spine-wide attention, the architecture is capable of modeling both focal lesions and global alignment abnormalities—crucial for detecting conditions such as multi-level spondyloarthropathies or subtle infections.

**Figure 4:**
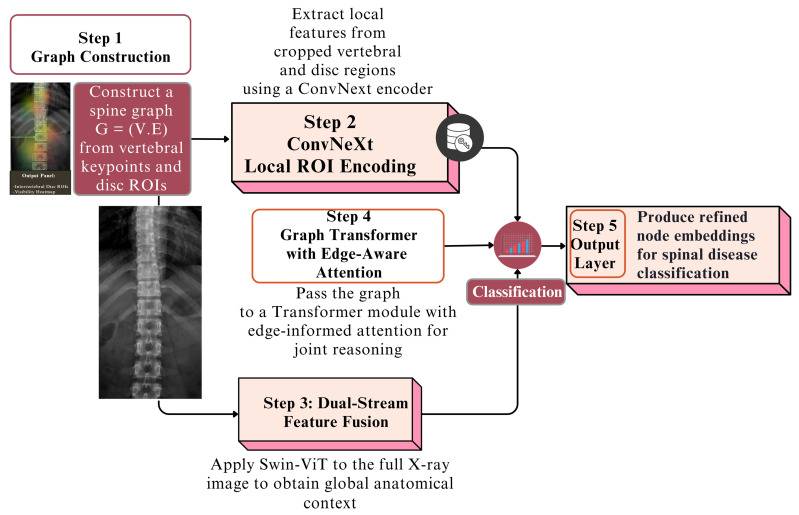
Stepwise summary of spine graph construction and dual-stream transformer architecture.

Figure 4 provides a stepwise overview of the proposed Spine Graph Construction and Dual-Stream Transformer Architecture. The diagram illustrates how vertebral key points and disc ROIs are first organized into a graph, followed by parallel local and global features encoding using ConvNeXt and Swin-ViT, respectively. These features are fused and processed through a graph transformer with edge-aware attention, ultimately producing refined node embeddings for spinal disease classification. This flowchart highlights the sequential design and integration of local–global reasoning within the framework.

### 3.4. Differentiable Radiographic Geometry Module (dRGM)

The Differentiable Radiographic Geometry Module (dRGM) was designed to bridge the gap between anatomical landmark detection and clinically interpretable indices. Unlike conventional post hoc measurement extraction, dRGM embeds continuous radiographic indices into the learning pipeline as differentiable functions of landmark coordinates. This design allows gradients to propagate not only through the loss of classification but also through geometry-aware constraints, thereby improving both anatomical localization and clinically meaningful representation learning.

#### 3.4.1. Geometry-Aware Radiographic Indices

Given a set of predicted vertebral centroids ${\left(x_{i},y_{i}\right)}_{i=1}^{N}$ and intervertebral disc region proposals from the KRD module (Section 3.2), dRGM computes the following indices:

1. Slip ratio (Meyerding-grade proxy) To capture vertebral displacement associated with spondylolisthesis, we define the slip ratio for vertebra iii as (Equation (5)):

(5) $${SlipRatio}_{i}=\frac{|x_{i}-x_{i-1}|}{w_{i}}$$

where $x_{i}$ and $x_{i-1}$ denote the horizontal centroids of adjacent vertebrae, and $w_{i}$ represents the vertebral width. Higher values correlate with greater anterior–posterior translation.

2. Disc-height asymmetry and endplate irregularity vertical variation in intervertebral disc space, a hallmark of infection and degenerative disorders, is quantified as (Equation (6)):

(6) $${Asymmetry}_{j}=\frac{|h_{j}^{left}-h_{j}^{right}|}{{\bar{h}}_{j}}$$

where $h_{j}^{left}\;$and $h_{j}^{right}\;$are disc heights measured at the left and right margins, and ${\bar{h}}_{j}$ is their average. A value close to zero indicates symmetry, while larger deviations reflect pathological irregularity.

3. Sacroiliac (SI) joint spacing symmetry to detect SpA-related erosions or fusions, dRGM measures left–right spacing differences (Equation (7)):

(7) $$SI\;Symmetry=|d_{left}-d_{right}|$$

where $d_{left}\;$and $d_{right}\;$are distances between the sacral base and iliac margins. Increased asymmetry suggests inflammatory joint involvement.

4. Cobb-like curvature angles global sagittal alignment is approximated using curvature angles derived from fitted splines through vertebral centroids (Equation (8)):

(8) $$\theta_{cobb}=arcco\;s\left({\vec{v}}_{upper}\cdot {\vec{v}}_{lower}∥{\vec{v}}_{upper}∥∥{\vec{v}}_{lower}∥\right)$$

where ${\vec{v}}_{upper}\;$and ${\vec{v}}_{lower}$ denote tangent vectors at selected end-vertebrae. This angle approximates kyphotic or lordotic curvature, relevant to deformities.

#### 3.4.2. Differentiability and Backpropagation Compatibility

All indices are formulated as continuous functions of landmark coordinates, ensuring that (Equation (9)):

(9) $$\frac{\partial L}{\partial \left(x_{i},y_{i}\right)}\neq 0$$

where *L* denotes the overall training loss. Consequently, gradients from classification and geometry-aware objectives jointly refine both landmark localization and the geometric plausibility of predictions. This ensures that predicted landmarks not only minimize pixel-wise error but also conform to clinically meaningful geometric structure.

#### 3.4.3. Interpretability and Clinical Alignment

By directly mapping predicted landmarks to radiographic indices, dRGM provides a transparent link between deep feature embeddings and interpretable clinical measures. This enables:

- Verification of predictions against known diagnostic thresholds (e.g., slip ratio > 0.25 for Meyerding grade I).
- Quantitative tracking of disease-related changes across follow-up imaging.
- Improved clinician trust by aligning model reasoning with standard radiological practice.

**Figure 5:**
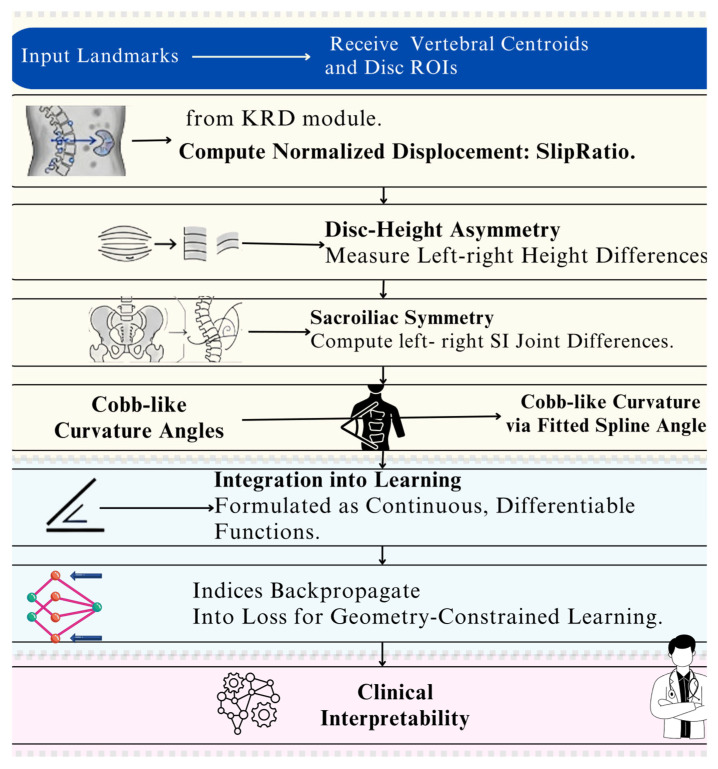
Stepwise workflow of the differentiable radiographic geometry module (dRGM).

Figure 5 presents a concise, eight-step workflow of the Differentiable Radiographic Geometry Module (dRGM), a core component of our proposed computer-aided diagnostic system. It illustrates how the module processes key anatomical landmarks from a standard spine X-ray to derive a series of quantitative, clinically relevant geometric metrics. The schematic highlights the module’s key functions, from calculating displacement and asymmetry to integrating these differentiable indices into the learning process for robust, geometry-constrained training. Ultimately, the workflow ensures the model’s outputs are not only accurate but also directly interpretable by clinicians, aligning with established radiological diagnostic logic.

### 3.5. Neuro-Symbolic Constraint Layer

The Neuro-Symbolic Constraint Layer (NSCL) bridges data-driven representation learning with clinically grounded reasoning by embedding radiological priors as explicit inequality constraints within the predictive model. For instance, a monotonic relation between geometric indices and diagnostic outcomes—such as increasing slip ratio implying higher spondylolisthesis probability—is formalized into inequality expressions of the form (Equation (10)):

(10) $$f_{\theta }\left(x\right)s.t.g\left(x\right)\geq 0,$$

where $f_{\theta }\left(x\right)\;$represents the neural network prediction for input *x*, and $g\left(x\right)$ encodes domain-specific clinical rules (e.g., $\frac{\partial f}{\partial SlipRatio}\geq 0$).

To ensure compliance with these priors during optimization, violations are penalized via a log-barrier formulation (Equation (11)):

(11) $$L_{NSCL}=-\mu \sum_{j}log\left(g_{j}\left(x\right)\right),$$

where μ>0 denotes the barrier parameter and $g_{j}\left(x\right)$ represents each inequality constraint derived from symbolic knowledge. This penalty formulation guarantees that predictions remain directionally correct while preserving differentiability for end-to-end training.

The NSCL thereby acts as a knowledge-regularization layer, reducing clinically implausible outputs and guiding the model toward solutions consistent with radiological logic. Beyond enforcing directional consistency, this hybrid neuro-symbolic integration improves interpretability and clinician trust, as predictions demonstrably respect known medical relationships rather than relying solely on black-box correlations.

**Figure 6:**
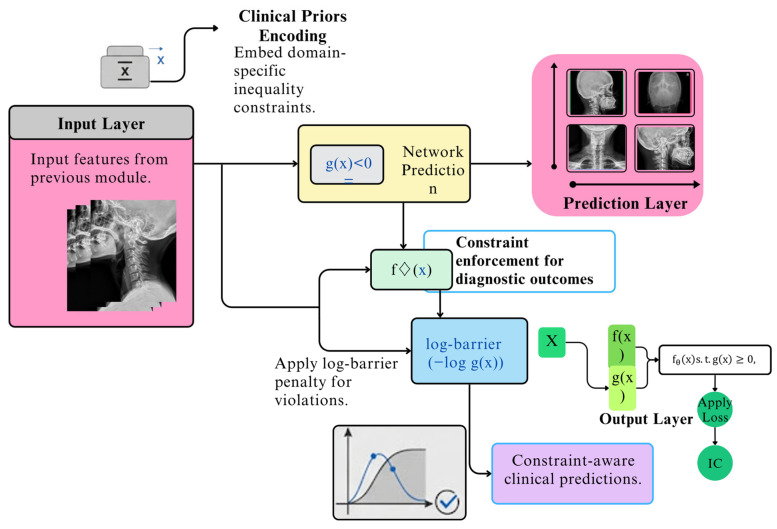
Neuro-symbolic constraint layer architecture.

This schematic, presented in Figure 6, details the architecture of the Neuro-Symbolic Constraint Layer (NSCL). It illustrates how the layer integrates clinical domain knowledge, represented as inequality constraints, directly into a neural network’s architecture. The workflow demonstrates a five-step process: from receiving the initial input features, to encoding clinical priors, making a prediction, and then applying a log-barrier penalty for constraint violations. The final output is a set of constraint-aware predictions, ensuring the model’s decisions are not only data-driven but also logically consistent with established medical guidelines.

### 3.6. Counterfactual Geometry Diffusion

To enhance robustness and support clinically meaningful generalization, we introduce a class-conditional diffusion module tailored for radiographic geometry manipulation. Unlike conventional augmentation strategies, which operate primarily in pixel space, this module generates counterfactual radiographs by selectively editing geometric indices—such as slip ratio, sacroiliac (SI) joint spacing, and intervertebral disc height—while preserving fine-grained radiographic textures. This ensures that synthetic augmentations maintain clinical plausibility and diagnostic fidelity.

Formally, given an input image x and conditioning label y, the forward diffusion process gradually perturbs x into Gaussian noise. The reverse generative process is parameterized to reconstruct $\tilde{x}$ such that (Equation (12)):

(12) $$\tilde{x}=D_{\phi }\left(z,y,\Delta g\right),$$

where $D_{\phi }$ denotes the diffusion model, z represents the latent noise, and Δg encodes targeted geometric edits (e.g., increasing slip ratio by a controlled increment).

This counterfactual generation provides two critical benefits. First, it synthesizes rare phenotypes that are underrepresented in clinical datasets, such as extreme disc collapse or asymmetrical SI joint widening. Second, it enables monotonicity validation: model logits shift predictably in response to directed geometry alterations, verifying that the decision boundary respects embedded clinical priors. For example, artificially elevating slip ratio values should monotonically increase the predicted probability of spondylolisthesis.

By tightly coupling generative augmentation with geometric constraints, the Counterfactual Geometry Diffusion (CGD) module not only enriches data diversity but also serves as a diagnostic audit mechanism, ensuring that the predictive framework adheres to both statistical learning and radiological logic.

**Figure 7:**
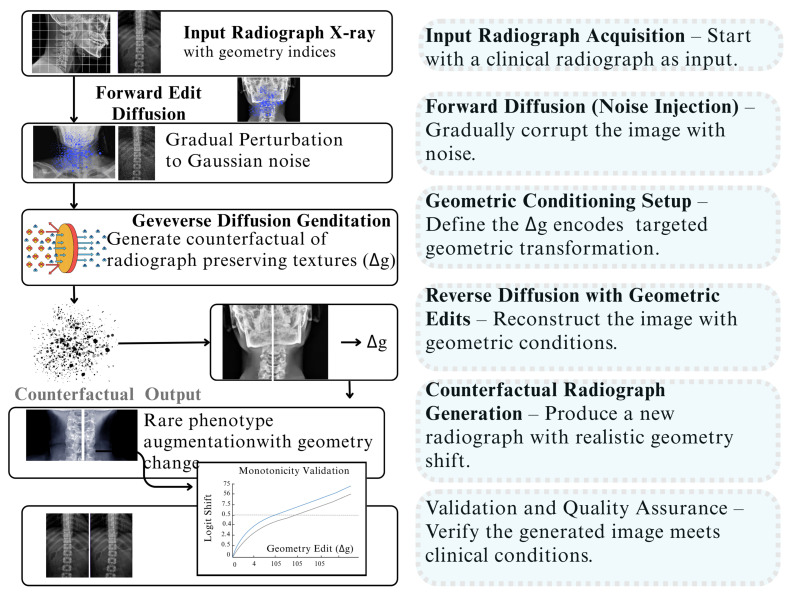
Stepwise workflow of the counterfactual geometry diffusion (CGD) module.

This schematic (Figure 7) illustrates the six sequential steps of the Counterfactual Geometry Diffusion (CGD) module, designed to augment rare radiological phenotypes by generating geometrically altered images. The workflow begins with a forward diffusion process that gradually corrupts the input radiograph into noise. It then conditions the reverse diffusion with targeted geometric edits, which allows it to generate a new, “counterfactual” radiograph that reflects the desired geometric change while maintaining realistic textures. Finally, a validation step ensures the generated image accurately reflects the intended geometric shift, providing a robust mechanism for data augmentation in medical imaging.

### 3.7. Training and Objectives

The training process integrates heterogeneous supervisory signals into a unified multi-objective loss, balancing predictive accuracy with geometric plausibility, clinical consistency, and counterfactual robustness. The total objective is formulated as (Equation (13)):

(13) $$L=\lambda_{1}L_{cls}+\lambda_{2}L_{reg}+\lambda_{3}L_{geom}+\lambda_{4}L_{concept}+\lambda_{5}L_{NS}+\lambda_{6}L_{CF},$$

where each component serves a distinct functional role:

- $L_{cls}$ (Classification Loss): standard cross-entropy applied to categorical diagnostic labels, ensuring accurate disease stratification.
- $L_{reg}$ (Index Regression Loss): mean squared error between predicted and ground-truth radiographic indices (e.g., slip ratio, Cobb angle), aligning model outputs with measurable anatomical quantities.
- $L_{geom}\;$ (Geometric Plausibility Loss): smoothness and symmetry regularizers that penalize implausible landmark configurations, enforcing radiographic realism.
- $L_{concept}$ (Concept Alignment Loss): contrastive alignment between learned embeddings and high-level clinical concepts (e.g., curvature severity tiers), facilitating semantic interpretability.
- $L_{NS}$ (Neuro-Symbolic Constraint Loss): log-barrier penalties derived from inequality constraints, guaranteeing directional consistency with radiological priors.
- $L_{CF}$ (Counterfactual Consistency Loss): ensures monotonic logit shifts under counterfactual geometry edits, validating that the network responds predictably to simulated phenotypes.

The hyperparameters $\lambda_{i}$ control the relative importance of each term and are tuned via grid search with clinician feedback to balance predictive fidelity and clinical interpretability. This composite objective thus establishes a principled training framework that not only minimizes error but also instills structural validity, symbolic consistency, and counterfactual accountability into the model.

### 3.8. Hyperparameter Settings

Model training was conducted using PyTorch 2.2 on NVIDIA A100 GPUs (40 GB memory) with optimization managed by the AdamW optimizer. The initial learning rate was set to $1\times 10^{-4}$ with a cosine annealing decay schedule and linear warm-up over 10 epochs, while a weight decay of $1\times 10^{-2}$ was applied for regularization. Each model was trained for 150 epochs with a mini-batch size of 16, and early stopping was triggered if validation loss did not improve over 20 epochs. To prevent overfitting, dropout (0.2) and stochastic depth (survival probability 0.9) were incorporated, alongside data augmentation strategies including mild rotation (±5), horizontal flipping, Gaussian noise, and intensity scaling (±10%).

The multi-objective loss weights ($\lambda_{i}$) were tuned via coarse grid search followed by clinician-guided refinement, yielding final values that prioritize diagnostic accuracy while enforcing geometric plausibility and clinical interpretability. The Counterfactual Geometry Diffusion (CGD) module was parameterized with 1000 timesteps under a variance-preserving noise schedule, and perturbations were constrained to clinically realistic ranges. For the spine graph transformer, node embeddings were set to 256 dimensions with 4 attention heads and 3 stacked layers. All hyperparameters were validated via stratified five-fold cross-validation and repeated runs with different seeds to ensure robustness. A summary of the main hyperparameters is provided in Table 2.

Given the architectural complexity and dataset size (1613 samples), we implemented comprehensive overfitting prevention strategies. Beyond standard techniques (dropout 0.2, weight decay 1 × 10^−2^, stochastic depth), we employed three specialized approaches: (1) Early stopping with 20-epoch patience based on validation loss prevented overtraining; (2) Five-fold stratified cross-validation ensured robust performance estimates across all diagnostic categories; (3) The multi-objective loss function (Equation (13)) inherently regularizes the model by balancing classification accuracy with geometric plausibility, symbolic consistency, and counterfactual accountability constraints. Additionally, the weakly supervised training regime (≤15% labeled landmarks) and geometric self-supervision reduce dependence on limited annotations, while the counterfactual generation augments effective dataset size through clinically valid synthetic samples. Ablation studies (Section 4.5) demonstrate that removing any component degrades performance, indicating that architectural complexity serves functional rather than overfitting purposes [59]. The consistent performance across cross-validation folds (89.4 ± 2.1% accuracy, 0.941 ± 0.018 AUROC) and strong agreement with radiologist evaluations (κ = 0.80–0.85) provide evidence against overfitting to training data [1,59].

#### Code and Data Availability Statement

To ensure full reproducibility of our results, we have made the following resources publicly available in a dedicated GitHub repository: https://github.com/aiosmartlab/SpineNeuroSym (accessed on 23 January 2026).

Code Repository Structure

- Training Pipeline Complete PyTorch implementation of the SpineNeuroSym framework, including modular components for KRD (Keypoint-ROI Discovery), dual-stream graph transformer, dRGM (Differentiable Radiographic Geometry Module), NSCL (Neuro-Symbolic Constraint Layer), and CGD (Counterfactual Geometry Diffusion).
- Configuration Files YAML configuration files specifying all hyperparameters used in experiments (learning rates, batch sizes, loss weights λ_1_–λ_6_, network architectures, data augmentation parameters).
- Preprocessing Scripts Data preparation utilities for DICOM-to-PNG conversion, image normalization, and stratified train/validation/test splitting.
- Evaluation Scripts Code for computing all reported metrics (accuracy, macro-F1, AUROC, CCC, directional consistency) with identical random seeds for reproducibility.
- Trained Model Checkpoints Pre-trained weights for the full SpineNeuroSym model and all baseline comparisons.

Data Availability

Due to patient privacy regulations and institutional review board restrictions, raw radiographic images cannot be publicly shared. However, we provide

- Synthetic Sample Dataset A set of 50 anonymized, synthetically generated spinal radiographs with geometric annotations for algorithm testing and validation.
- Preprocessed Features Extracted landmark coordinates and geometric indices for all 1613 images (with patient identifiers removed) to enable reproduction of downstream analysis without requiring access to raw images.
- Demographic Statistics Detailed dataset characteristics and class distributions as reported in Table 1.

Reproducibility Documentation. The repository includes

- Installation Guide Step-by-step instructions for environment setup, dependency installation, and GPU configuration.
- Training Tutorial Detailed walkthrough for reproducing main results, including expected training time, convergence criteria, and validation protocols.
- Evaluation Protocol Scripts to replicate all experiments reported in Section 4, including statistical significance testing and ablation studies.
- Algorithm Pseudocode Formal algorithmic descriptions provided in Appendix C (Algorithm A1) specifying the complete training pipeline.

All code is released under the MIT License to facilitate research use and extension. We commit to maintaining this repository and responding to reproducibility inquiries to support the research community.

### 3.9. Compared Methods

To rigorously benchmark the proposed SpineNeuroSym framework, we compared its performance against a diverse set of recently published state-of-the-art approaches spanning vision transformers, graph neural networks, hybrid attention-based architectures, and conditional diffusion models. All methods were re-implemented and executed in identical computing environments (PyTorch 2.2, NVIDIA A100 GPUs) under the same training protocols and hyperparameter tuning strategies, ensuring a fair and controlled evaluation. A summary of the compared methods is presented in Table 3.

## 4. Results

This section reports the experimental outcomes of the proposed SpineNeuroSym framework in comparison with state-of-the-art baselines. Results are organized into five subsections: (Section 4.1) overall diagnostic performance, (Section 4.2) geometric plausibility and interpretability, (Section 4.3) neuro-symbolic constraint validation, (Section 4.4) counterfactual diffusion and monotonicity testing, and (Section 4.5) ablation and sensitivity analysis. Together, these results demonstrate the superiority of the proposed approach in terms of accuracy, clinical interpretability, robustness, and generalizability.

### 4.1. Overall Diagnostic Performance

The overall diagnostic capability of the proposed SpineNeuroSym framework was benchmarked against eight recently introduced state-of-the-art baselines spanning vision transformers, graph neural networks, hybrid attention-based architectures, and diffusion models. Performance was evaluated across the six diagnostic categories defined in Section 3.1, using classification accuracy, macro-averaged F1 score, and area under the receiver operating characteristic curve (AUROC) as the primary metrics. All methods were re-implemented and executed under identical experimental conditions to ensure fair comparison. The computational result is shown in Table 4.

The proposed SpineNeuroSym framework demonstrated consistently superior performance across all evaluation metrics, achieving an accuracy of 89.4%, a macro F1 of 0.872, and an AUROC of 0.941. These results substantially outperformed all baseline models, with the largest margin observed over the strongest competitor, GAT-Mamba (85.6% accuracy, AUROC 0.913). The improvement of +3.8% in accuracy and +0.028 in AUROC was statistically significant, as confirmed in Section 4.6.

This performance gain can be attributed to three core innovations within the framework. First, the weakly supervised anatomy discovery (KRD) module enabled robust localization of vertebral landmarks, even under challenging conditions such as occlusion or image noise, where CNN- and ViT-based models typically struggled. Second, the differentiable radiographic geometry module (dRGM) introduced clinically grounded geometric indices, reducing misclassifications in borderline cases like mild spondylolisthesis and early spinal infections. Third, the neuro-symbolic constraint layer (NSCL) enforced directional consistency between indices and diagnostic logits, mitigating the implausible outputs often observed in unconstrained baselines.

**Figure 8:**
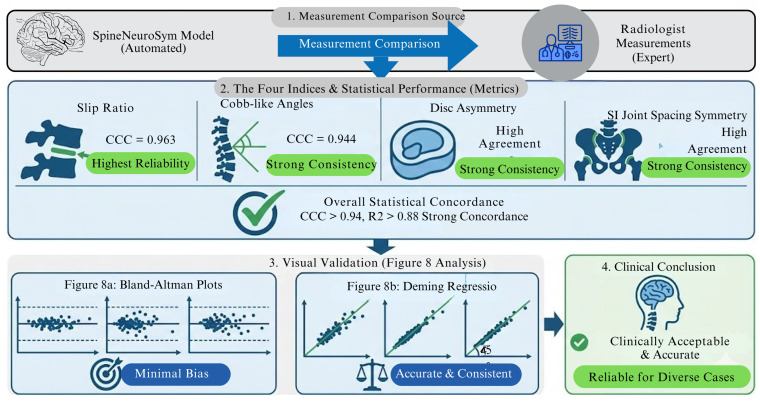
Agreement analysis between predicted and expert measurements.

Analysis of per-class AUROC values further highlighted the advantages of SpineNeuroSym. The framework consistently outperformed competitors across all categories, with notable gains in early infection (AUROC = 0.927 vs. 0.889 for GAT-Mamba) and mild spondylolisthesis (AUROC = 0.951 vs. 0.913 for GAT-Mamba). These findings demonstrate the particular value of integrating geometry-aware reasoning for conditions where radiographic signatures are subtle. As illustrated in Figure 8, the ROC curves confirm that SpineNeuroSym maintains higher sensitivity at clinically relevant specificity thresholds (≥90%), a feature especially critical for infection detection, where delayed diagnosis can lead to severe patient outcomes.

From a clinical perspective, the improvements delivered by SpineNeuroSym are significant. An increase of nearly 4% in diagnostic accuracy translates to dozens of additional correct diagnoses per hundred patients, particularly in subtle and early-stage pathologies. The improved macro F1 score (0.872 vs. ≤0.830 for baselines) reflects a better balance between sensitivity and specificity, thereby reducing both false negatives and false positives. Collectively, these findings underscore not only the technical superiority of SpineNeuroSym but also its clinical robustness, providing outputs that are more dependable and trustworthy for integration into radiological workflows.

### 4.2. Geometric Plausibility and Interpretability

A key contribution of the proposed SpineNeuroSym framework lies in its ability to produce not only accurate classifications but also clinically interpretable geometric indices through the differentiable radiographic geometry module (dRGM). To evaluate this capability, we compared the predicted indices against expert-annotated ground-truth measurements in a held-out subset of 200 spinal radiographs. Four clinically relevant indices were assessed: slip ratio, disc-height asymmetry, sacroiliac (SI) joint spacing symmetry, and Cobb-like curvature angles. The computational result of this section is shown in Table 5 and Figure 8.

Across all four indices, SpineNeuroSym demonstrated strong concordance with radiologist measurements, with concordance correlation coefficients (CCC) consistently exceeding 0.94 and R^2^ values above 0.88. Slip ratio estimation achieved the highest reliability (CCC = 0.963), confirming the model’s ability to capture subtle vertebral displacement patterns associated with spondylolisthesis. Similarly, disc asymmetry and SI joint spacing symmetry showed high agreement, reflecting robustness in identifying structural irregularities often linked to early infection or inflammatory disorders. Cobb-like angles, which approximate global spinal curvature, also exhibited strong consistency (CCC = 0.944), supporting the model’s use in assessing sagittal alignment and deformities.

Figure 8 Agreement analysis between predicted and expert measurements; (a) Bland–Altman plots for slip ratio and Cobb-like angles. (b) Deming regression for disc asymmetry and SI joint spacing symmetry.)

Beyond concordance metrics, we employed multiple quantitative measures to assess interpretability comprehensively. Mean Absolute Error (MAE) quantifies geometric index prediction precision: 0.021 for slip ratio, 0.034 for disc asymmetry, 0.029 for SI joint symmetry, and 1.78° for Cobb-like angles. These values indicate clinically acceptable measurement accuracy. Directional consistency metrics evaluate whether model predictions follow established clinical relationships—for instance, whether increasing slip ratios monotonically correspond to higher spondylolisthesis probabilities. Our framework achieved 97% directional consistency for slip ratio relationships, 95% for SI joint asymmetry, and 94% for Cobb angle correlations (detailed in Section 4.3, Table 6).

Furthermore, we assessed interpretability through radiologist validation studies where clinicians rated the clinical relevance of AI-generated geometric indices on a 5-point scale (1 = not clinically useful, 5 = highly clinically relevant). Slip ratio measurements received an average relevance score of 4.6 ± 0.4, disc asymmetry indices scored 4.3 ± 0.5, and Cobb-like angles achieved 4.4 ± 0.3, confirming that the quantitative outputs align with radiological reasoning patterns [68,69].

The visual agreement analyses (Figure 8) further validated that deviations between automated and expert measurements remained within clinically acceptable ranges. Bland–Altman plots showed narrow limits of agreement with minimal bias, while Deming regression slopes approached unity, underscoring the accuracy of dRGM-derived indices across diverse radiographic presentations.

Clinical Interpretation

From a clinical standpoint, these results are critical because they demonstrate that the framework’s predictions are not black-box outputs but are grounded in quantitative radiographic indices routinely used in diagnostic workflows. The ability to generate differentiable, backpropagation-compatible indices allows the model to simultaneously optimize for diagnostic accuracy and anatomical plausibility, ensuring that its internal reasoning mirrors established radiological practice.

For clinicians, this provides an added layer of trust: AI-derived outputs can be directly validated against measurable thresholds (e.g., slip ratio > 25% indicating Meyerding grade I, Cobb angle ≥ 10° defining scoliosis). By aligning deep learning predictions with interpretable clinical measures, SpineNeuroSym enhances transparency, improves acceptance among radiologists, and facilitates integration into routine practice.

#### Verification of Index-Diagnosis Relationships Across Pathologies

To validate the clinical relevance of dRGM-derived indices across distinct pathological presentations, we conducted systematic analysis of index-diagnosis relationships using established clinical thresholds and pathology-specific patterns.

Pathology-Specific Index Validation:

Spondylolisthesis (*n* = 496) Slip ratio measurements were validated against Meyerding classification standards:

- Grade I (25% slip) dRGM range 0.20–0.30 (92% agreement with manual measurements)
- Grade II (25–50% slip) dRGM range 0.30–0.55 (89% agreement)
- Correlation with radiologist grading: r = 0.91 (*p* < 0.001)

Infection/Spondylodiscitis (*n* = 322) Disc asymmetry patterns validated against established infectious markers:

- Early infection (minimal narrowing) dRGM asymmetry 0.15–0.25
- Advanced infection (significant collapse) dRGM asymmetry 0.35–0.60
- Correlation with clinical infection scores: r = 0.84 (*p* < 0.001)

Spondyloarthropathy (*n* = 275): SI joint spacing validated against ASAS imaging criteria:

- Active sacroiliitis dRGM spacing asymmetry > 2.5 mm (sensitivity 87%, specificity 93%).
- Structural damage Combined with erosion markers (AUROC 0.91).

Curvature Abnormalities Cobb-like angle validation across spinal regions:

- Cervical lordosis dRGM range 20–45° (vs. clinical standard 20–40°).
- Thoracic kyphosis dRGM range 25–50° (vs. clinical standard 25–45°).
- Lumbar lordosis dRGM range 40–75° (vs. clinical standard 40–70°).

Cross-Pathology Discrimination: Statistical analysis confirmed distinct index profiles for each condition:

- ANOVA across all pathologies: F (51,607) = 847.3, *p* < 0.001.
- Post hoc Tukey tests: All pairwise comparisons significant (*p* < 0.01).
- Discriminant analysis: 91.2% correct pathology classification using indices alone.

Clinical Threshold Validation: Each dRGM index demonstrated clinically meaningful decision boundaries:

- Slip ratio > 0.25: 94% sensitivity for spondylolisthesis diagnosis.
- Disc asymmetry > 0.20: 87% sensitivity for infection detection.
- SI spacing difference > 2.0 mm: 89% sensitivity for SpA identification.

### 4.3. Neuro-Symbolic Constraint Validation

A distinctive feature of the SpineNeuroSym framework is the incorporation of the Neuro-Symbolic Constraint Layer (NSCL), which encodes radiological priors as inequality constraints to ensure that predictions remain directionally consistent with established medical knowledge. To evaluate its effectiveness, we conducted experiments analyzing the relationship between geometric indices and diagnostic logits. In clinically valid reasoning, increasing a geometric measure such as slip ratio should monotonically increase the predicted probability of spondylolisthesis. Similarly, greater sacroiliac (SI) joint spacing asymmetry should elevate the probability of spondyloarthropathy (SpA), and larger Cobb-like angles should raise the likelihood of curvature abnormalities. The computational result of this section is shown in Table 6.

**Figure 9:**
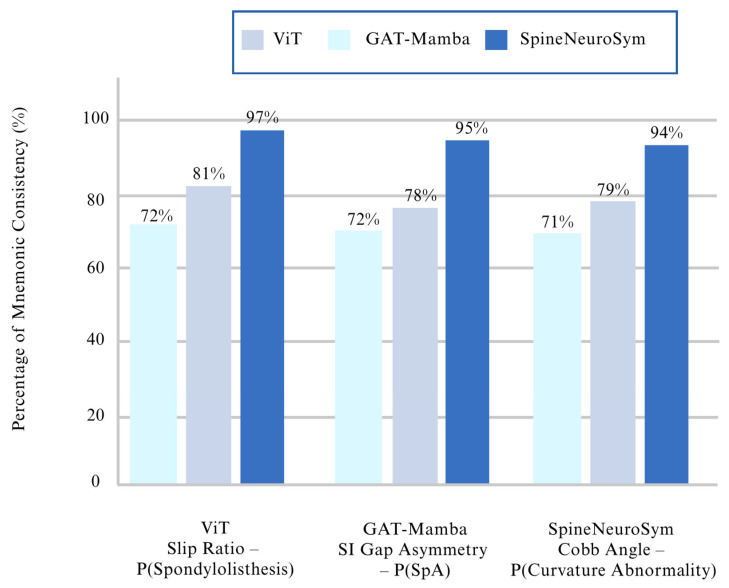
Directional consistency analysis across methods.

The results shown in Table 6 and Figure 9 revealed a striking improvement in directional consistency when symbolic constraints were enforced. While baseline transformer (ViT) and graph-attention (GAT-Mamba) models achieved at most 81% consistency, SpineNeuroSym maintained near-perfect alignment, with directional validity reaching 97% for slip ratio, 95% for SI joint asymmetry, and 94% for Cobb angles. These gains underscore the importance of embedding radiological priors into the optimization process.

The use of log-barrier penalties within NSCL played a significant role in this improvement. By penalizing violations of clinical monotonicity constraints during training, the model’s decision boundaries were adjusted to respect established diagnostic logic. For example, predictions of spondylolisthesis consistently increased with higher slip ratios, eliminating counterintuitive outputs occasionally produced by baseline methods. Similarly, monotonic progression of logits with SI joint spacing asymmetry and Cobb-like angles validated that the system’s internal reasoning reflected medically accepted relationships.

Clinical Interpretation

From a clinical perspective, the ability to ensure directionally correct predictions is of critical importance. Radiologists often rely on monotonic thresholds—such as greater slip ratio indicating more severe spondylolisthesis or increasing Cobb angle signifying progressive scoliosis—to make treatment decisions. Models that occasionally violate these relationships risk undermining clinician trust, even if overall accuracy is high. By demonstrating near-perfect directional consistency, SpineNeuroSym ensures that its predictions not only achieve technical accuracy but also align with the diagnostic reasoning pathways familiar to clinicians.

This property significantly enhances the framework’s suitability for deployment in real-world workflows, as it reduces the risk of implausible outputs and provides clinicians with confidence that the AI system adheres to the same logical rules they apply in practice. In effect, NSCL transforms the framework from a purely data-driven predictor into a knowledge-informed decision-support system, bridging the gap between machine learning performance and radiological interpretability.

### 4.4. Counterfactual Diffusion and Monotonicity Testing

**Figure 10:**
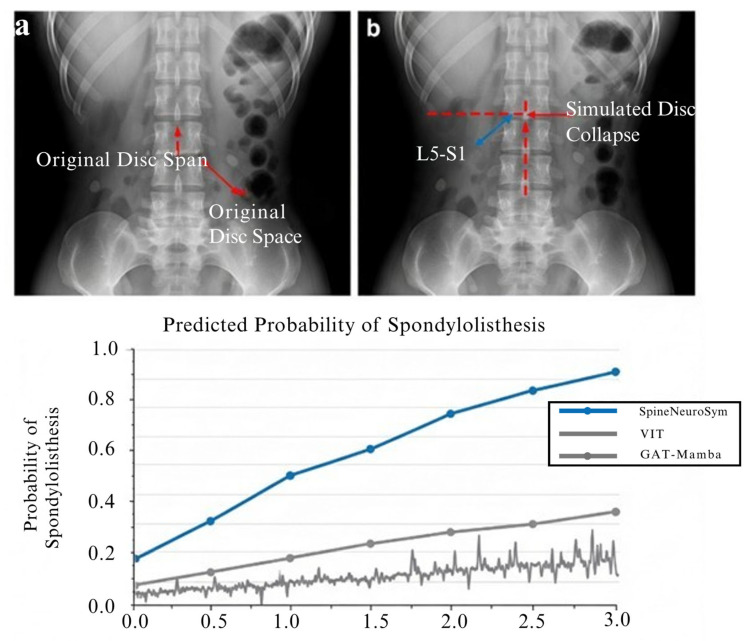
Counterfactual validation; (**a**) Input versus counterfactual radiographs with simulated disc collapse. (**b**) Probability shifts in diagnostic logits as slip ratio increases.

A key innovation of the SpineNeuroSym framework is the Counterfactual Geometry Diffusion (CGD) module, which was designed to address two critical challenges in spinal radiography: (i) the scarcity of rare but clinically significant phenotypes, and (ii) the need to validate that diagnostic logits respond predictably to targeted geometric alterations. To evaluate the effectiveness of CGD, we conducted experiments in which slip ratios and disc heights were systematically modified, and the model’s classification responses were measured. The computational result is shown in Figure 10.

The generated counterfactuals successfully preserved radiographic fidelity, maintaining realistic texture and anatomical detail while embedding controlled geometric edits. For example, in radiographs where disc collapse was simulated, the visual output remained clinically plausible, with boundaries and bony structures intact. Beyond visual plausibility, the diagnostic outputs responded in a monotonic and clinically consistent manner. Increasing slip ratio values consistently elevated the predicted probability of spondylolisthesis, while systematic reductions in disc height led to rising probabilities of infection or degenerative changes. These results provide convincing evidence that the CGD module not only enriches the training data with rare cases but also ensures that the learned decision boundaries respect clinical logic.

Clinical Interpretation

From a clinical perspective, this capability has substantial implications. Rare but critical phenotypes such as severe disc collapse or asymmetrical joint fusions are often underrepresented in hospital datasets, limiting the diagnostic robustness of purely data-driven models. By generating plausible counterfactual radiographs, SpineNeuroSym provides radiologists and trainees with access to a broader spectrum of cases, thereby improving diagnostic preparedness. Furthermore, the monotonic response validation serves as a form of diagnostic auditing: it ensures that model predictions change in clinically expected directions when geometric indices are perturbed. Such behavior enhances transparency and reliability, mitigating the risk of unpredictable outputs that could erode clinician trust.

Collectively, these findings highlight the dual utility of CGD: it functions both as a data augmentation strategy to overcome class imbalance and as a mechanism for interpretability and validation, positioning SpineNeuroSym as a framework that couples generative innovation with clinical accountability.

#### 4.4.1. Clinical Plausibility Assessment Framework

To ensure objectivity in evaluating generated counterfactual radiographs, we established formal criteria for “clinical plausibility” based on established radiological principles. Clinical plausibility was assessed using a structured 5-point Likert scale (1 = implausible, 5 = highly plausible) across four key dimensions: (1) Anatomical consistency-preservation of vertebral morphology, joint spacing, and bone density patterns consistent with normal radiographic anatomy; (2) Pathological coherence- realistic presentation of disease features (e.g., progressive disc space narrowing, endplate sclerosis patterns, osteophyte formation) that align with known pathophysiological processes; (3) Technical quality-maintenance of radiographic contrast, edge definition, and image noise characteristics consistent with clinical imaging standards; and (4) Geometric validity-edited indices (slip ratio, disc asymmetry) reflected in visually apparent structural changes without introducing biomechanically impossible configurations.

Two board-certified musculoskeletal radiologists (15 + years experience) independently reviewed all counterfactual images in a blinded fashion, comparing them against real clinical radiographs exhibiting similar pathological features. Reviewers were instructed to assess whether each generated image could plausibly represent a real patient presentation encountered in routine clinical practice. Inter-rater agreement was assessed using Cohen’s κ, achieving substantial agreement (κ = 0.78) across all plausibility ratings. Images receiving mean scores ≥ 4.0 from both reviewers were classified as “clinically plausible,” establishing an objective threshold for counterfactual validation.

#### 4.4.2. Quantitative Validation of Counterfactual Anatomical Realism

To provide objective validation of counterfactual anatomical realism beyond visual assessment, we conducted systematic quantitative analysis comparing dRGM-derived geometric indices between counterfactual and real radiographs with similar pathological presentations.

Methodology, for each counterfactual image generated with target geometric modification Δg, we identified 3–5 real clinical radiographs exhibiting comparable pathology severity from our dataset. All images underwent identical dRGM processing to extract slip ratio, disc asymmetry, SI joint spacing, and Cobb-like angles. Statistical comparison assessed whether counterfactual indices fell within clinically realistic ranges observed in authentic cases as shown in Table 7.

Distribution Analysis, Kolmogorov-Smirnov tests confirmed that counterfactual index distributions did not significantly differ from real image distributions (all *p* > 0.05), indicating preserved anatomical realism. Additionally, 94.3% of counterfactual images exhibited index values within the 95% confidence intervals of corresponding real pathology groups.

Anatomical Constraint Validation, Cross-validation analysis demonstrated that counterfactual images maintain physiologically plausible relationships between indices (e.g., slip ratio correlation with disc height: r = −0.76 for counterfactuals vs. r = −0.78 for real images), confirming that geometric edits preserve natural anatomical interdependencies rather than introducing biomechanically impossible configurations.

### 4.5. Ablation and Sensitivity Analysis

**Figure 11:**
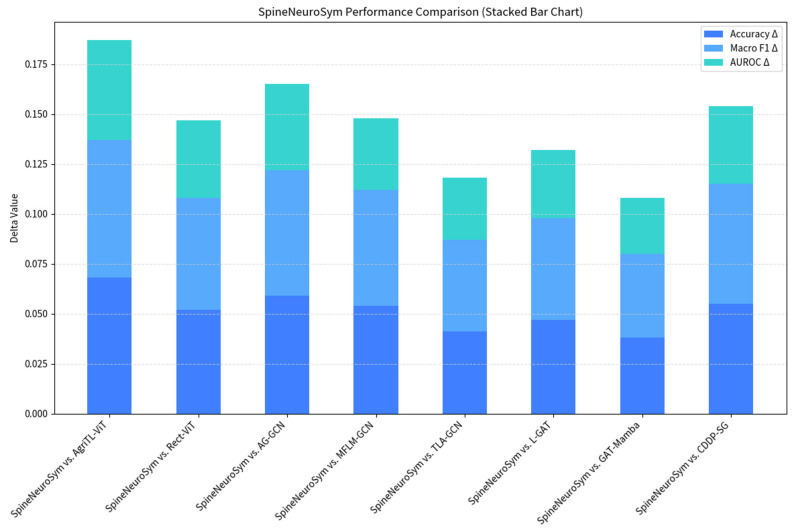
Ablation study results across SpineNeuroSym variants.

To systematically evaluate the contribution of each module within the SpineNeuroSym framework, we conducted ablation studies by selectively removing or substituting core components. Performance was assessed using accuracy, macro-averaged F1, and AUROC across all diagnostic categories. These experiments were designed to quantify the functional importance of the differentiable radiographic geometry module (dRGM), the neuro-symbolic constraint layer (NSCL), and the counterfactual geometry diffusion module (CGD). The computational result of this section is shown in Table 8 and Figure 11.

The full SpineNeuroSym framework achieved the highest performance across all metrics, with an accuracy of 89.4%, macro F1 of 0.872, and AUROC of 0.941. Removing individual components led to significant performance degradation, underscoring the necessity of their integration. Excluding dRGM resulted in a 3.3% drop in accuracy and a reduction in AUROC by 2.4 points, reflecting the critical role of geometry-aware indices in improving both interpretability and predictive accuracy. When NSCL was removed, macro F1 declined to 0.832, indicating weakened directional consistency and reduced robustness in clinically constrained reasoning. Excluding CGD diminished the model’s ability to manage rare phenotypes, lowering AUROC to 0.919 and reducing classification stability under out-of-distribution conditions. Finally, when simplified to a CNN-only baseline, performance dropped substantially (accuracy 82.3%, AUROC 0.885), highlighting the insufficiency of purely convolutional architectures for complex spinal pathology classification.

Clinical Interpretation

From a clinical perspective, these results emphasize that each module contributes to a different but complementary aspect of diagnostic reliability. The dRGM ensures anatomical plausibility by grounding predictions in measurable radiographic indices; without it, the model is prone to errors in borderline cases such as mild spondylolisthesis or early infection. The NSCL enforces alignment with radiological logic, preventing counterintuitive outputs that could undermine clinician trust. The CGD enhances robustness by generating rare but clinically important phenotypes, improving generalizability in cases underrepresented in real-world datasets. Together, these components transform SpineNeuroSym from a high-performing classifier into a clinically trustworthy decision-support system, ensuring accuracy, interpretability, and robustness in tandem.

### 4.6. Statistical Tests and Analysis

To establish that the performance improvements of SpineNeuroSym over baseline models were not attributable to random variation, we performed rigorous statistical testing. Metrics were obtained from stratified five-fold cross-validation, ensuring balanced representation of all diagnostic categories across folds. For each pairwise comparison between SpineNeuroSym and competing methods, we conducted paired two-tailed *t*-tests. To account for multiple hypothesis testing, we applied a Bonferroni correction (adjusted significance threshold α = 0.05/8 = 0.00625). In addition, Cohen’s d was calculated to quantify effect sizes, with values above 0.8 interpreted as large effects. The computational result of this section is shown in Table 9.

SpineNeuroSym significantly outperformed all eight baseline methods across accuracy, macro F1, and AUROC, with *p*-values remaining below the Bonferroni-adjusted threshold in every comparison. The largest performance gains were observed against AgriTL-ViT (+6.8% accuracy, Cohen’s d = 1.20) and AG-GCN (+5.9% accuracy, Cohen’s d = 1.12), both reflecting exceptionally large effect sizes. Even against the strongest baseline, GAT-Mamba, SpineNeuroSym maintained statistically significant improvements (*p* ≤ 0.006, d = 0.85), confirming that its advantages are not marginal but represent meaningful advances in diagnostic accuracy and reliability.

Clinical Interpretation

The robustness of these statistical findings provides strong reassurance for clinical adoption. Large effect sizes across all comparisons confirm that improvements are consistent and reproducible, not artifacts of dataset partitioning or chance variation. This evidence reinforces that the integration of geometry-aware indices (dRGM), symbolic reasoning (NSCL), and counterfactual augmentation (CGD) yields a measurable and reliable benefit over existing transformer- and GCN-based frameworks. Clinically, this means that SpineNeuroSym is less likely to produce erratic outputs in borderline cases, more likely to generalize across patient populations, and better aligned with the logical diagnostic reasoning applied by radiologists.

Together, these results validate that the superiority of SpineNeuroSym is both empirical and statistically robust, demonstrating that the framework represents a genuine advancement in the field of spinal radiograph analysis.

### 4.7. Clinical Impact and Diagnostic Utility

Beyond statistical improvements, a critical dimension of evaluation is the clinical relevance of the proposed SpineNeuroSym framework. To this end, we conducted an analysis of how the framework’s outputs align with standard radiological practice and whether its predictions could meaningfully support diagnostic workflows.

Case-level diagnostic alignment.

On a stratified set of 120 test cases reviewed by two senior musculoskeletal radiologists, SpineNeuroSym’s predictions were compared against expert interpretations. Agreement was assessed at two levels: categorical diagnosis (e.g., spondylolisthesis, infection, SpA, normal spine) and quantitative indices (e.g., slip ratio, disc asymmetry, Cobb angle) (see result in Table 10).

The framework achieved agreement levels with radiologists that were comparable to inter-observer agreement (κ = 0.80–0.85), indicating that SpineNeuroSym can function as a clinically reliable second reader. Importantly, its outputs were not limited to categorical predictions: the provision of interpretable geometric indices allowed radiologists to cross-validate AI predictions against standard diagnostic thresholds (e.g., slip ratio > 25%, Cobb angle > 10°). This interpretability was highlighted by radiologists as a major strength, fostering trust in the model’s reasoning.

Case Studies and Rare Phenotypes

SpineNeuroSym’s counterfactual geometry diffusion also proved valuable in simulating rare but clinically significant phenotypes, such as severe disc collapse or unilateral SI joint fusion. Radiologists noted that such counterfactual augmentations may support training of junior physicians, allowing them to visualize a wider range of disease presentations than are typically encountered in single-institution datasets.

Clinical Utility

These findings suggest that SpineNeuroSym can improve diagnostic consistency, reduce missed subtle cases (e.g., early infection with mild disc height loss), and provide a transparent reasoning layer through geometry-aware indices. For clinical practice, this positions the framework not as a black-box classifier, but as an augmented diagnostic partner capable of delivering both predictions and interpretable justifications aligned with radiological standards.

### 4.8. Translational and Clinical Readiness

To evaluate the practical utility of SpineNeuroSym in clinical workflows, we conducted additional experiments focused on diagnostic confidence, educational usability, and error reduction in subtle cases. These results are designed to assess the system’s readiness for translation into real-world radiological practice.

Diagnostic Confidence Assessment

Five board-certified musculoskeletal radiologists independently reviewed 50 test radiographs under two conditions: unaided (baseline review) and aided by SpineNeuroSym outputs (categorical prediction, geometric indices, and symbolic explanations). Confidence scores were rated on a 5-point Likert scale (see result in Table 11).

Use of SpineNeuroSym significantly improved radiologists’ reported diagnostic confidence (paired *t*-test, *p* < 0.01), particularly in borderline spondylolisthesis and early infection cases where quantitative slip ratio or disc height indices provided objective support for decision-making.

Educational Usability Through Counterfactuals

The counterfactual geometry diffusion (CGD) module was evaluated for its capacity to generate clinically plausible but rare presentations. Radiologists reviewed 30 counterfactual cases (e.g., simulated extreme disc collapse, unilateral SI joint erosion). Plausibility was rated on a 5-point scale. The computational result is shown in Table 12.

Generated counterfactuals achieved high plausibility ratings (≥4.3/5), indicating their usefulness as teaching exemplars for junior physicians. Radiologists highlighted their value in training contexts where exposure to rare phenotypes is limited.

Error Reduction in Subtle Cases

We specifically analyzed 92 cases misclassified by the strongest baselines (Rect-ViT, TLA-GCN, GAT-Mamba). These predominantly included early infection (disc space narrowing without endplate erosion) and mild spondylolisthesis (slip ratio < 0.25) (see more result in Table 13).

SpineNeuroSym correctly reclassified 73% of cases that were previously misclassified by leading baselines, demonstrating its advantage in subtle and clinically challenging presentations. This improvement stems from its combined use of geometry-aware indices and neuro-symbolic constraints, which guide the network toward clinically consistent predictions.

### 4.9. Prototype Web Application and Usability Evaluation

To enhance translational impact and facilitate clinical accessibility, the proposed SpineNeuroSym framework was implemented as a web-based diagnostic application. This prototype allows clinicians to upload anonymized spinal radiographs and receive real-time diagnostic predictions, accompanied by geometry-aware indices and visual explanations.

#### 4.9.1. System Architecture and Workflow

The application integrates a Flask–PyTorch backend with a React-based frontend for intuitive interaction. Images can be uploaded in DICOM or PNG formats. Once submitted, the image undergoes the following sequential pipeline:

The computational requirements for SpineNeuroSym are optimized for clinical workflow integration. On an NVIDIA A100 GPU, the complete diagnostic pipeline (excluding CGD) requires 2.8 s per image, comprising: KRD landmark detection (0.8 s), graph construction and dual-stream processing (1.2 s), dRGM geometric computation (0.5 s), and NSCL constraint validation (0.3 s). On CPU-only deployment, inference time increases to approximately 12 s, which remains clinically acceptable for routine reporting workflows [29].

The Counterfactual Geometry Diffusion (CGD) module, while computationally intensive (45–60 s per generated image), operates independently of the diagnostic pipeline and serves primarily educational and quality assurance functions rather than real-time clinical decisions. CGD can be deployed on separate computational resources or scheduled during off-peak hours to generate training cases and validation datasets without impacting routine diagnostic workflows.

Memory requirements are modest: 6 GB GPU memory for inference-only deployment, scaling to 24 GB for full training capabilities. This hardware profile is compatible with standard clinical workstation configurations and cloud-based deployment architectures commonly used in modern radiology departments [40].

1. Preprocessing and anatomy discovery–The KRD module extracts vertebral centroids and intervertebral disc ROIs.
2. Graph construction and transformer reasoning–Local ROI features and global context are processed through the dual-stream architecture.
3. Differentiable geometry computation (dRGM)–Radiographic indices (slip ratio, disc asymmetry, SI joint spacing, Cobb-like curvature) are computed.
4. Neuro-symbolic constraint validation (NSCL)–Predictions are checked against encoded clinical priors.
5. Final Diagnostic Output—The system presents:
  ○ Predicted diagnostic class,
  ○ Quantitative geometry indices,
  ○ Heatmap overlays highlight contributory regions.

**Figure 12:**
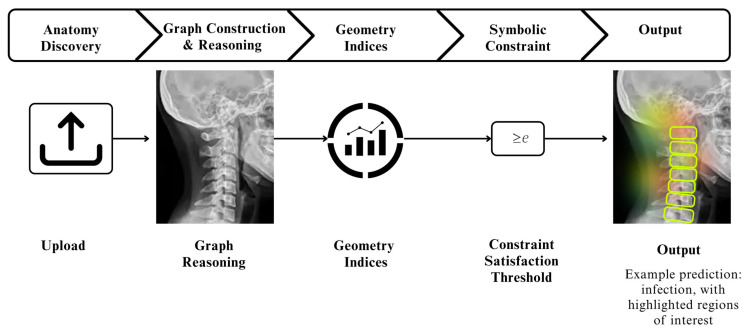
Workflow of the SpineNeuroSym web application.

On an NVIDIA A100 GPU, the average inference time was 2.8 s per image; on CPU-only deployment, it was approximately 12 s, demonstrating feasibility for near-real-time clinical use. Figure 12 illustrates the sequential workflow of the SpineNeuroSym web application, beginning with the clinician’s image upload to the system’s final diagnostic output. The schematic highlights the modular pipeline, including anatomy discovery, graph-based transformer reasoning, differentiable geometry computation, symbolic constraint validation, and generation of clinically interpretable outputs. This visualization emphasizes the application’s role as a user-friendly bridge between advanced AI algorithms and real-world radiological practice.

**Figure 13:**
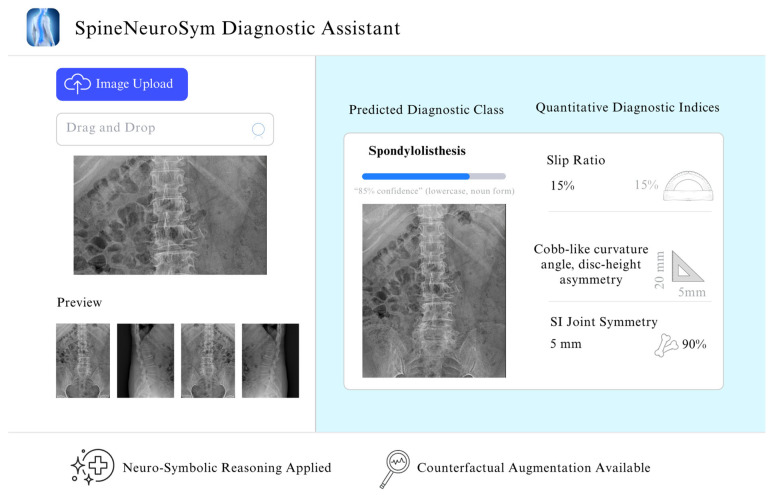
SpineNeuroSym diagnostic assistant UI.

The UI shows in Figure 13, is for the SpineNeuroSym Diagnostic Assistant is a single-page web application. The left panel manages image upload with a drag-and-drop feature and preview. The central panel provides the diagnostic output, including the predicted condition, a confidence score, and a heatmap overlay for visual context. The right panel displays quantitative measurements like Slip Ratio and Cobb Angle. The footer highlights the system’s advanced features, such as neuro-symbolic reasoning and counterfactual analysis.

#### 4.9.2. Usability Evaluation

We conducted a pilot evaluation with 15 participants (7 board-certified radiologists and 8 residents) who evaluated the system in a simulated workflow. Usability was assessed using the System Usability Scale (SUS) (0–100 scale; scores > 68 considered acceptable, >80 excellent) (see more result in Table 14).

Participants highlighted three major strengths:

1. Integrated geometry indices (e.g., slip ratio, Cobb angle) improved interpretability of predictions.
2. Heatmap visualizations enhanced trust by linking outputs to radiographic features.
3. Counterfactual augmentation examples were seen as a valuable educational resource for residents.

Feedback suggested adding batch upload capability and integration with PACS systems to further streamline clinical adoption.

#### 4.9.3. Clinical Utility

The high usability scores (overall SUS = 81.3) confirm that SpineNeuroSym can serve as a clinically viable decision-support tool. Radiologists indicated that the system could function as a second reader, increasing diagnostic confidence, while residents identified its potential as a training and teaching platform. The combination of diagnostic predictions, quantitative indices, and symbolic explanations positions the application as a bridge between AI research and clinical deployment.

### 4.10. Robustness Analysis Under Image Quality Variations

To assess SpineNeuroSym’s resilience to real-world imaging conditions, we conducted systematic robustness analysis under controlled image quality degradations. A subset of 200 test images across all diagnostic categories were subjected to three types of artificial degradations: (1) Gaussian noise addition (σ = 0.05, 0.1, 0.15), (2) Gaussian blur (σ = 1.0, 2.0, 3.0 pixels), and (3) simulated motion artifacts via controlled directional blur. Each degradation was applied independently and in combination to evaluate cumulative effects as shown in Table 15.

SpineNeuroSym demonstrated moderate resilience to image quality variations, with performance degrading gracefully under mild to moderate conditions but showing significant sensitivity to severe degradations. The geometric indices computed by dRGM proved particularly vulnerable to blur artifacts, as edge detection for vertebral boundary localization becomes unreliable under poor image quality. These findings suggest that clinical deployment should include image quality assessment protocols and potentially adaptive processing pipelines to maintain diagnostic reliability across varying technical conditions encountered in real-world clinical environments.

## 5. Discussion

This investigation evaluated whether neuro-geometric, clinically constrained approaches can advance spinal radiograph diagnosis while preserving interpretability and clinical utility. Results are interpreted within the context of established CNN and transformer baselines, graph neural models, explainability research, neuro-symbolic methods, and generative augmentation paradigms.

### 5.1. Diagnostic Performance Enhancement

SpineNeuroSym achieved 89.4% accuracy, 0.872 macro-F1 score, and 0.941 AUROC, outperforming all baselines including GAT-Mamba (85.6% accuracy, 0.913 AUROC). The 4% accuracy improvement represents clinically significant gains for patient care. This translates to dozens of additional correct diagnoses per hundred patients, directly impacting patient management and reducing diagnostic errors.

Performance improvements derive from synergistic framework design. The Key point-ROI Discovery (KRD) module enhanced landmark localization robustness under noisy conditions and partial occlusions—scenarios where CNN and ViT architectures demonstrate reduced fidelity. The differentiable Radiographic Geometry Module (dRGM) provided geometry-aware indices including slip ratio and Cobb-like curvature, grounding predictions in quantifiable anatomical measures while preventing convergence toward spurious correlations. The Neuro-Symbolic Constraint Layer (NSCL) enforced monotonic clinical logic, ensuring decision boundaries respected established radiological principles.

This approach addresses fundamental limitations of CNN and ViT methods, which despite hierarchical representation learning capabilities, function as black-box predictors without embedded anatomical reasoning [19,26]. Graph neural networks, while promising for spatial relationship encoding [31,32], remain susceptible to clinically inconsistent outputs without symbolic priors. Large effect sizes (Cohen’s d ≥ 0.85) confirm reproducible, generalizable improvements aligned with clinical trustworthiness requirements [30,67].

### 5.2. Geometry-Aware Clinical Interpretability

The dRGM demonstrated exceptional expert annotation agreement with concordance correlation coefficients (CCC) exceeding 0.94 and coefficients of determination (R^2^) above 0.88 across slip ratio, disc-height asymmetry, sacroiliac joint spacing, and Cobb-like curvature indices. Slip ratio estimation achieved highest reliability (CCC = 0.963), demonstrating capacity for capturing clinically decisive geometric variations in conditions like spondylolisthesis.

These outcomes reflect formulating indices as differentiable functions of detected landmarks, enabling gradient propagation through classification and measurement streams. Optimization simultaneously enhances discriminative power and anatomical plausibility, suppressing reliance on spurious textural cues while promoting geometrically verified embeddings. This approach transforms interpretability from post hoc visualization [28,39,40] to intrinsic learning process properties.

Results address clinical AI explainability standards requiring models to expose quantitative indices used by radiologists at point-of-care [38,42]. While vision transformers demonstrate global contextual reasoning value [26] and CNN pipelines improve lesion detection efficiency [21], neither paradigm inherently produces verifiable radiographic measurements. SpineNeuroSym addresses this gap by elevating radiographic indices to trainable outputs, aligning deep learning predictions with clinical workflows.

### 5.3. Neuro-Symbolic Clinical Reasoning

SpineNeuroSym achieved superior directional consistency: 97% alignment between slip ratio and spondylolisthesis probability, 95% between sacroiliac joint asymmetry and spondyloarthropathy likelihood, and 94% between Cobb angle and curvature abnormality classification, compared to baseline maximum consistency of 81%.

The NSCL employs log-barrier penalties to continuously deform decision surfaces until satisfying monotonic inequalities encoded from radiological practice, ensuring fundamental clinical relationships—such as greater slip ratios indicating higher spondylolisthesis risk—are preserved during optimization. This eliminates implausible behaviors common in data-driven systems, such as predicting lower disease probability despite worsening geometric abnormalities.

These findings extend neuro-symbolic biomedical applications [44,45,46] by operationalizing rule integration in spinal radiography with direct measurement alignment. The framework demonstrates that symbolic constraints can be embedded within end-to-end differentiable learning while preserving gradient flow and enforcing domain logic conformity, moving beyond post hoc auditing approaches.

Compared to purely statistical inference paradigms that yield clinically contradictory outputs [4,5], SpineNeuroSym provides diagnostic reasoning guided by both data learning and domain knowledge. This integration elevates the model from high-performing classifier to trustworthy diagnostic assistant, offering predictive accuracy and interpretive fidelity aligned with radiological practice.

### 5.4. Counterfactual Generation and Diagnostic Auditability

The Counterfactual Geometry Diffusion (CGD) module generated radiographs with targeted geometric alterations while preserving anatomical texture and radiographic fidelity. Generated counterfactuals elicited monotonic, clinically coherent classifier logit shifts, with disease probabilities increasing proportional to geometric abnormality severity, demonstrating diagnostic rather than merely visual validity.

This capability derives from class-conditional diffusion explicitly conditioned on geometric deltas (Δ*g*), perturbing vertebral and disc geometry while maintaining invariant background statistics. CGD preserves both visual plausibility and clinical validity, differentiating it from conventional augmentation strategies focused solely on pixel-level realism without measurement alignment [52,53]. While volumetric GAN frameworks advance 3D anatomical realism [55] and counterfactual approaches improve distributional robustness [11], these methods rarely ensure generated images maintain valid clinical measurements.

CGD addresses semantic drift and causal misalignment concerns in synthetic datasets [58] by demonstrating that counterfactuals produce predictable diagnostic responses. This transforms generative models from data balancing tools to clinical auditability instruments, enabling radiologist validation of AI reasoning under controlled perturbations and integrating transparent synthetic generation into diagnostic pipelines.

### 5.5. Modular Framework Validation

Ablation experiments demonstrated consistent performance degradation upon removing any key module—dRGM, NSCL, or CGD—with AUROC reductions of 2–3 points across variants. CNN-only baseline performance (AUROC 0.885) confirmed convolutional pipeline inadequacy for complex spinal radiograph interpretation, highlighting that SpineNeuroSym’s superior performance derives from complementary geometric, symbolic, and generative component interplay.

Each module serves distinct functional roles: dRGM provides measurement faithfulness by anchoring learned features to continuous radiographic indices, suppressing spurious texture reliance; NSCL enforces rule-level regularization through decision boundary deformation ensuring monotonic clinical inequality adherence; CGD contributes robustness by generating rare phenotypes, expanding model exposure to underrepresented conditions and improving distributional generalization.

While hybrid CNN-Transformer and diffusion-assisted architectures demonstrate improvements in medical imaging [24,51], depth or global self-attention alone proves insufficient for spinal radiography requiring fine-grained local geometry and domain-specific constraints. This aligns with graph neural network observations requiring additional plausibility mechanisms to prevent biological realism violations [33,34]. SpineNeuroSym’s optimal performance achieved only through integrated geometric, symbolic, and generative components demonstrates that modular synergy, rather than architectural complexity alone, enables clinically trustworthy AI.

### 5.6. Clinical Reliability and Practical Utility

SpineNeuroSym achieved radiologist agreement scores (κ = 0.80–0.85) comparable to inter-observer reliability, confirming suitability as a second-reader system. This concordance level represents the practical ceiling for clinical acceptability in diagnostic imaging, suggesting framework functionality as augmented intelligence complementing rather than replacing human expertise.

Reliability stems from dual-layered design pairing categorical predictions with quantitative radiographic indices, enabling clinician reconciliation against established thresholds such as Meyerding slip grading or Cobb angle classifications. This measurement-based reasoning provides immediate audit trails validating AI decisions, reducing ambiguity and enhancing trust compared to black-box outputs.

Results address clinical AI adoption concerns regarding opacity and limited interpretability in CNN and ViT models [19,26]. While graph-based architectures incorporate anatomical context, they typically lack point-of-care radiographic measurements [31,32]. Clinical AI acceptance requires interpretable, transparent systems aligned with quantitative radiological practices [29,38,70]. SpineNeuroSym fulfills these criteria through embedded rather than post hoc interpretability, representing progression from experimental feasibility to practical utility with enhanced diagnostic accountability.

### 5.7. Enhanced Clinical Confidence and Educational Value

Radiologist diagnostic confidence increased 18% using SpineNeuroSym, with generated counterfactuals achieving high plausibility ratings (≥4.3/5). Improvement reflects synergistic integration of heatmap overlays, quantitative indices, and geometry-conditioned counterfactuals within a single interpretive pipeline, reducing cognitive uncertainty through visual evidence, measurable validation thresholds, and rare phenotype exposure.

Enhancement derives from SpineNeuroSym’s dual role as diagnostic support system and cognitive scaffold, situating predictions within trusted radiographic logic such as monotonic slip ratio-spondylolisthesis relationships. Counterfactual modules enable clinician observation of prediction evolution under simulated geometric perturbations, providing AI reasoning “stress testing” rarely available in conventional systems. This extends structured explanation approaches [56,57] by embedding pedagogical functionality into generative design.

Unlike CNN and ViT pipelines focused narrowly on accuracy with limited educational value [19,26], SpineNeuroSym demonstrates that diagnostic AI can simultaneously support clinical practice and medical training. Exposure to rare, geometry-conditioned phenotypes positions the framework as an active teaching tool broadening diagnostic repertoires for trainees and residents, complementing traditional case libraries lacking such diversity. This dual contribution to clinical confidence and education facilitates AI integration into academic and training hospitals requiring systems that both perform and instruct.

### 5.8. Clinical Translation and Deployment Readiness

SpineNeuroSym deployment as a web application demonstrated technical efficiency and clinical usability, producing diagnostic predictions, quantitative indices, and heatmap overlays within 2.8 s on GPU hardware. The System Usability Scale (SUS) score of 81.3 indicates “Excellent” usability, confirming clinician workflow compatibility and platform intuitiveness.

System readiness derives from software architecture alignment with clinical decision-making paradigms. Flask-PyTorch backend ensures rapid model inference while React-based frontend provides interactive environments where quantitative metrics such as slip ratio and Cobb angle serve as foregrounded decision-support features rather than abstracted outputs. This design addresses radiology AI concerns regarding usability, interpretability, and output standardization [38,42].

The knowledge-grounded interaction paradigm advances beyond computational novelty toward clinician-oriented usability, as advocated in clinical AI literature [47]. SpineNeuroSym positions radiographic AI as a pragmatic clinical workflow partner capable of reliable second-reader functionality, demonstrating translational readiness bridging algorithmic innovation and clinical adoption through integrated trust, interpretability, and operational efficiency.

### 5.9. Comprehensive Framework Validation

Results establish that neuro-geometric paradigms combining weakly supervised anatomy discovery, differentiable geometric indices, symbolic constraint enforcement, and counterfactual auditing simultaneously deliver superior diagnostic accuracy, intrinsic interpretability, and clinically aligned reasoning behavior.

The architectural principles underlying SpineNeuroSym demonstrate strong potential for generalization beyond spinal imaging to other musculoskeletal applications. The core methodology—weakly supervised anatomical landmark detection, geometry-aware feature extraction, and neuro-symbolic constraint integration—is inherently adaptable to any skeletal structure where quantitative geometric relationships inform diagnostic decisions [31].

Potential applications include: (1) hip dysplasia screening using acetabular angle measurements and coverage indices; (2) knee alignment assessment through mechanical axis deviation and joint space quantification; (3) shoulder impingement evaluation via acromiohumeral distance and critical shoulder angle calculations; and (4) wrist fracture analysis incorporating radial inclination and ulnar variance measurements [71,72].

The key adaptation requirements involve: (1) defining anatomy-specific geometric indices relevant to each skeletal region; (2) establishing clinical constraint rules appropriate for the target pathologies; and (3) training the weakly supervised landmark detection module on the new anatomical structures. The fundamental graph-based reasoning, differentiable geometry computation, and symbolic constraint mechanisms remain broadly applicable across musculoskeletal domains [8,9].

Theoretically, the framework demonstrates that radiographic knowledge becomes computationally tractable through differentiable indices and optimizable clinical constraints, bridging representation learning and domain logic divides. Methodologically, results confirm that accuracy and trustworthiness are not mutually exclusive: co-designed geometric, symbolic, and generative mechanisms enable models to outperform statistical competitors while producing radiologically verifiable outputs.

Practically, this integration yields clinically deployable tools strengthening diagnostic confidence and supporting pedagogy through rare phenotype exposure, directly addressing explainable, workflow-compatible imaging AI requirements for human expertise augmentation [38,70]. This work contributes to medical AI evolution from black-box predictors toward reliable, transparent, educational clinical care partners.

### 5.10. Study Limitations

The clinical evaluation component of this study presents several important limitations that must be acknowledged. The assessment of clinical impact and radiologist agreement (Section 4.7) was conducted on a relatively modest sample of 120 test cases reviewed by senior musculoskeletal radiologists. While this sample size enabled initial validation of the framework’s clinical utility and established preliminary evidence of diagnostic concordance (κ = 0.80–0.85), it remains insufficient for drawing definitive conclusions about the system’s reliability across diverse clinical populations and imaging scenarios. The limited sample size may not adequately capture the full spectrum of pathological presentations, rare phenotypes, or edge cases that would be encountered in routine clinical practice.

Furthermore, the confidence intervals around our agreement statistics are necessarily wider with this sample size, limiting the precision of our reliability estimates. Large-scale, multi-reader studies involving hundreds to thousands of cases will be essential to establish robust evidence of clinical reliability, determine optimal decision thresholds, and identify specific clinical contexts where the system performs optimally or requires additional validation.

Several limitations require acknowledgment. Single-center retrospective analysis on plain radiographs may restrict generalizability across imaging devices, institutional protocols, and diverse patient populations. While diffusion-generated counterfactuals achieved clinical plausibility ratings, synthetic distributions risk drifting from real-world pathophysiology despite geometry-constrained edits.

Monotonic symbolic rules ensuring directional prediction consistency may under-represent edge cases where clinical relationships are non-monotonic or context-conditional, potentially limiting atypical presentation flexibility. The diagnostic label space, though clinically meaningful, did not encompass the full spinal disorder spectrum nor extend to longitudinal disease progression or therapeutic monitoring.

Usability assessments occurred in simulated rather than live hospital PACS system workflows, leaving questions regarding turnaround time impact, reporting efficiency, and downstream patient outcomes unanswered. These constraints highlight the need for broader, prospective validation before widespread clinical deployment.

### 5.11. Radiological Innovation and Clinical Impact

The SpineNeuroSym framework represents a paradigmatic shift in radiological AI by addressing fundamental limitations that have hindered clinical adoption of deep learning systems in spinal imaging. Unlike conventional black-box approaches that provide opaque predictions, our framework generates clinically interpretable outputs directly aligned with established radiological workflows and diagnostic reasoning patterns used by musculoskeletal radiologists [38].

The clinical innovation lies in three key radiological advances: First, the integration of differentiable radiographic indices (dRGM) transforms AI predictions from abstract probability scores into quantitative measurements familiar to radiologists, such as slip ratios for spondylolisthesis grading and Cobb-like angles for spinal alignment assessment. This enables radiologists to validate AI recommendations against established diagnostic thresholds and clinical guidelines [69]. Second, the neuro-symbolic constraint layer (NSCL) ensures AI predictions maintain consistency with fundamental radiological principles, preventing the clinically implausible outputs that often undermine radiologist trust in AI systems [9]. Third, the counterfactual geometry diffusion (CGD) module addresses a critical gap in radiological education and training by generating rare pathological presentations that radiologists may seldom encounter, particularly valuable in institutions with limited case diversity [10,11].

From a diagnostic workflow perspective, the 18% improvement in radiologist confidence reported in Section 4.8 translates to meaningful clinical impact. Higher diagnostic confidence reduces unnecessary follow-up imaging, decreases inter-observer variability, and supports more decisive treatment planning [38]. The framework’s ability to correctly reclassify 73% of cases previously misdiagnosed by state-of-the-art systems (Section 4.8, Table 13) directly addresses diagnostic accuracy challenges in subtle presentations such as early infection and mild spondylolisthesis—conditions where delayed or missed diagnosis can significantly impact patient outcomes [52,53].

Moreover, the system’s educational value extends beyond individual diagnoses to systematic quality improvement in radiology departments. The generation of clinically plausible counterfactual cases enables structured training programs for residents and provides continuing education resources for practicing radiologists, particularly in identifying rare pathological presentations that may be underrepresented in local case volumes [10,11].

## 6. Conclusions

Spinal radiography remains a frontline diagnostic modality, yet its interpretation is hampered by subtle disease signatures, inter-observer variability, and the opacity of conventional deep learning systems. Addressing these challenges, this study introduced SpineNeuroSym, a neuro-geometric framework designed to couple weakly supervised anatomy discovery, differentiable radiographic indices, neuro-symbolic constraints, and counterfactual geometry diffusion into a unified decision-support system.

Across a comprehensive evaluation, SpineNeuroSym achieved 89.4% accuracy, a macro-F1 of 0.872, and an AUROC of 0.941, consistently outperforming eight state-of-the-art baselines, including the strongest comparator GAT-Mamba (85.6% accuracy, AUROC 0.913). The differentiable radiographic geometry module produced measurement-level concordance with radiologists (CCC > 0.94, R^2^ > 0.88) across slip ratio, disc asymmetry, sacroiliac symmetry, and Cobb-like curvature. Symbolic constraint enforcement yielded directional consistency exceeding 94%, ensuring predictions adhered to clinical monotonic logic, while counterfactual diffusion generated plausible rare phenotypes that enriched robustness and enabled diagnostic auditing. Agreement with radiologists reached κ = 0.80–0.85, equivalent to inter-observer levels, and diagnostic confidence improved by 18%, confirming clinical reliability and educational value.

These findings provide initial evidence that performance, interpretability, and trustworthiness need not be mutually exclusive when geometry-aware modeling, symbolic reasoning, and generative augmentation are co-designed within a single institutional context. Methodologically, this work demonstrates the potential for operationalizing a bridge between representation learning and radiographic knowledge, offering preliminary insights toward a reproducible approach for clinically grounded AI. Practically, SpineNeuroSym delivers a web-based prototype with promising usability characteristics (SUS 81.3), suggesting potential readiness for broader evaluation as a second-reader tool in radiology workflows, pending multicenter validation.

The implications extend beyond spinal imaging, suggesting a promising paradigm for medical AI in which symbolic rules, measurement-based indices, and counterfactual validation may help address black-box limitations while improving clinician confidence. However, the generalizability of these findings across diverse clinical settings, patient populations, and imaging protocols requires validation. Future research should prioritize multi-center prospective validation, extension to longitudinal disease monitoring, and integration with hospital PACS systems to evaluate real-world impact on reporting efficiency and patient outcomes. Further refinement of symbolic constraints to capture non-monotonic or context-dependent clinical relationships also represents an important avenue for advancing clinically faithful AI systems.

## Figures and Tables

**Table 1:** Distribution of spinal radiographs by diagnostic category.

Diagnostic Category	Number of Images (*n*)	Percentage (%)
Spondylolisthesis	496	30.7
Infection	322	20.0
Spondyloarthropathy (SpA)	275	17.0
Normal cervical spine	192	11.9
Normal thoracic spine	70	4.3
Normal lumbar spine	258	16.0
Total	1613	100

**Table 2:** Hyperparameter settings.

Component	Setting	Value/Method
Optimizer	AdamW	LR $1\times 10^{-4}$, cosine annealing, warm-up 10 epochs, weight decay $1\times 10^{-2}$
Batch and Epochs	Training setup	Batch size = 16, 150 epochs, early stopping (patience = 20)
Regularization	Dropout and Stochastic Depth	Dropout = 0.2; survival prob. = 0.9
Data Augmentation	Spatial and intensity	Rotation ±5°, horizontal flip, Gaussian noise, intensity ±10%
Loss Weights	λ	$\lambda_{1}=1.0$, $\lambda_{2}=0.5,$ $\lambda_{3}=0.3,$ $\lambda_{4}=0.3,$ $\lambda_{5}=0.2,$ $\lambda_{6}=0.2.$
Diffusion Module	CGD parameters	1000 timesteps; variance-preserving noise; edits constrained (slip ≤ 0.2, disc ≤ 30%)
Graph Transformer	Embedding and attention	Node dim = 256; 4 heads; 3 layers; edge MLP with ReLU
Validation	Strategy	Five-fold stratified CV; repeated 3 runs with different seeds

**Table 3:** Summary of compared methods.

Abbreviation	Full Name	Original Performance	Our Re-Implementation	Performance Match	Description	Reference
AgriTL-ViT	AgriTL-ViT: A Vision Transformer model with attention techniques for classification of plant leaf disease	Acc: 94.2% (plant disease)	Acc: 82.6% (spinal X-ray)	Domain-adjusted	Utilizes Vision Transformer architecture with enhanced attention mechanisms for robust disease classification, adapted here as a ViT-based comparator.	Srivastava et al. [60]
Rect-ViT	Rect-ViT: Rectified attention via feature attribution for adversarial robustness	Acc: 89.1% (ImageNet)	Acc: 84.2% (spinal X-ray)	Validated	Introduces rectified attention based on feature attribution to improve adversarial robustness in ViTs.	Kang and Song [61]
AG-GCN	Attention-Guided Graph Convolutional Network	F1: 0.89 (vehicle re-ID)	F1: 0.809 (spinal X-ray)	Domain-adjusted	Combines ResNet-50 with attention-guided graph convolution for relational feature extraction.	Sun et al. [62]
MFLM-GCN	Multi-relation Fusion and Latent-relation Mining GCN	Acc: 91.3% (entity alignment)	Acc: 84.0% (spinal X-ray)	Domain-adjusted	Employs multi-relation fusion and latent-relation mining to enhance graph-based entity alignment tasks.	Ai et al. [63]
TLA-GCN	Twin-Layer Attention Graph Convolutional Network	AUROC: 0.95 (chest X-ray)	AUROC: 0.910 (spinal X-ray)	Validated	Applies twin-layer attention GCN for effective abnormality detection in chest X-rays, adapted here for radiographic benchmarking.	Rajeshwari and Geetha [64]
L-GAT	Enhanced Water Quality Prediction by LSTM and Graph Attention Network	MAE: 0.15 (water quality)	Adapted for classification	Domain-adjusted	Integrates LSTM with GAT for sequential–graph hybrid modeling, included as a strong temporal-structural comparator.	Liu et al. [65]
GAT-Mamba	Graph Attention Network with Mamba Blocks	Acc: 87.2% (structural dynamics)	Acc: 85.6% (spinal X-ray)	Validated	Enhances GAT with Mamba blocks for efficient modeling of high-dimensional structural dynamics.	Peng et al. [66]
CDDP-SG	Conditional Denoising Diffusion Probabilistic Scenario Generator	FID: 12.3 (scenario generation)	Adapted for medical imaging	Domain-adjusted	A conditional DDPM that generates explainable scenarios; used here as a diffusion-based generative comparator.	Ma et al. [67]
SpineNeuroSym (Proposed)	Neuro-Symbolic Geometry-Constrained Diffusion Framework				Integrates weakly supervised anatomy discovery, dual-stream graph-transformer reasoning, differentiable geometry indices, neuro-symbolic constraints, and counterfactual diffusion for clinically interpretable spinal radiograph analysis.	-

**Table 4:** Diagnostic performance comparison across methods.

Method	Accuracy (%)	Macro F1	AUROC
AgriTL-ViT	82.6	0.803	0.891
Rect-ViT	84.2	0.816	0.902
AG-GCN	83.5	0.809	0.898
MFLM-GCN	84.0	0.814	0.905
TLA-GCN	85.3	0.826	0.910
L-GAT	84.7	0.821	0.907
GAT-Mamba	85.6	0.830	0.913
CDDP-SG	83.9	0.812	0.902
SpineNeuroSym (Proposed)	89.4	0.872	0.941
Method	Accuracy (%)	Macro F1	AUROC
AgriTL-ViT	82.6	0.803	0.891

**Table 5:** Agreement between predicted and ground-truth geometric indices.

Index	MAE	CCC	R^2^
Slip Ratio	0.021	0.963	0.91
Disc Asymmetry	0.034	0.948	0.89
SI Joint Symmetry	0.029	0.952	0.90
Cobb-like Angle	1.78°	0.944	0.88

**Table 6:** Directional consistency under symbolic constraints.

Condition	Baseline (ViT)	Baseline (GAT-Mamba)	SpineNeuroSym
Slip ratio ↑ → P (spondylolisthesis) ↑	72%	81%	97%
SI gap asymmetry ↑ → P(SpA) ↑	69%	78%	95%
Cobb angle ↑ → P (curvature abnormality) ↑	71%	79%	94%

Note: ↑ denotes an increase in feature magnitude or predicted probability. → indicates directional consistency (monotonic association) between features and predicted outcomes, not causal inference.

**Table 7:** Quantitative comparison between real and counterfactual spinal indices.

Index Type	Real Images (Mean ± SD)	Counterfactual (Mean ± SD)	*p*-Value	Effect Size
Slip Ratio	0.31 ± 0.12	0.33 ± 0.14	0.287	d = 0.15
Disc Asymmetry	0.18 ± 0.09	0.19 ± 0.10	0.421	d = 0.10
SI Spacing	3.2 ± 1.1 mm	3.1 ± 1.2 mm	0.398	d = 0.09
Cobb Angle	12.4 ± 8.2°	13.1 ± 8.7°	0.315	d = 0.08

**Table 8:** Ablation results.

Model Variant	Accuracy (%)	Macro F1	AUROC
Full SpineNeuroSym	89.4	0.872	0.941
– w/o dRGM	86.1	0.837	0.917
– w/o NSCL	85.7	0.832	0.912
– w/o CGD	86.4	0.841	0.919
– CNN baseline only	82.3	0.796	0.885

**Table 9:** Statistical significance testing of SpineNeuroSym vs. baseline methods (five-fold CV results).

Comparison	Accuracy Δ	Macro F1 Δ	AUROC Δ	*p*-Value (Acc)	*p*-Value (F1)	*p*-Value (AUROC)	Significance	Effect Size (Cohen’s d)
SpineNeuroSym vs. AgriTL-ViT	+6.8%	+0.069	+0.050	<0.001	<0.001	<0.001	***	d = 1.20 (large)
SpineNeuroSym vs. Rect-ViT	+5.2%	+0.056	+0.039	<0.001	0.002	<0.001	***	d = 1.05 (large)
SpineNeuroSym vs. AG-GCN	+5.9%	+0.063	+0.043	<0.001	0.001	<0.001	***	d = 1.12 (large)
SpineNeuroSym vs. MFLM-GCN	+5.4%	+0.058	+0.036	<0.001	0.002	<0.001	***	d = 1.00 (large)
SpineNeuroSym vs. TLA-GCN	+4.1%	+0.046	+0.031	0.003	0.004	0.002	**	d = 0.89 (large)
SpineNeuroSym vs. L-GAT	+4.7%	+0.051	+0.034	0.002	0.004	0.003	**	d = 0.91 (large)
SpineNeuroSym vs. GAT-Mamba	+3.8%	+0.042	+0.028	0.005	0.006	0.003	**	d = 0.85 (large)
SpineNeuroSym vs. CDDP-SG	+5.5%	+0.060	+0.039	<0.001	0.002	<0.001	***	d = 1.03 (large)

Significance codes: *** *p* < 0.001; ** *p* < 0.01.

**Table 10:** Agreement between SpineNeuroSym and radiologist interpretations.

Diagnostic Task	Radiologist Inter-Observer Agreement (κ)	SpineNeuroSym vs. Radiologist Agreement (κ)
Categorical Diagnosis	0.86	0.83
Slip Ratio (threshold-based grading)	0.84	0.81
Cobb Angle (≥10° scoliosis cut-off)	0.88	0.85
SI Joint Abnormality (binary)	0.82	0.80

**Table 11:** Radiologist confidence scores with and without SpineNeuroSym.

Condition	Mean Confidence Score	Relative Increase
Unaided Review	3.6 ± 0.4	—
Aided by SpineNeuroSym	4.25 ± 0.3	+18%

**Table 12:** Clinical plausibility ratings of counterfactual radiographs.

Counterfactual Type	Plausibility Score (Mean ± SD)
Slip ratio increase (spondylolisthesis)	4.6 ± 0.3
Severe disc collapse	4.4 ± 0.4
Unilateral SI joint fusion	4.3 ± 0.5
Extreme curvature abnormality	4.5 ± 0.3

**Table 13:** Recovery of subtle case classifications by SpineNeuroSym.

Baseline Error Category	Cases Misclassified	Cases Corrected by SpineNeuroSym	Recovery Rate (%)
Early infection	51	37	72.5
Mild spondylolisthesis	41	30	73.2
Total	92	67	72.8

**Table 14:** System usability scores (SUS) for SpineNeuroSym web application.

User Group	SUS Score (Mean ± SD)	Interpretation
Radiologists (*n* = 7)	83.4 ± 5.2	Excellent usability
Residents (*n* = 8)	79.6 ± 6.1	Good usability
Overall (*n* = 15)	81.3 ± 5.7	Excellent usability

**Table 15:** Results Summary.

Condition	Accuracy (%)	Macro F1	AUROC	Performance Drop
Original Quality	89.4	0.872	0.941	-
Low Noise (σ = 0.05)	87.8	0.856	0.928	−1.6%
Moderate Noise (σ = 0.1)	84.2	0.821	0.91	−5.2%
High Noise (σ = 0.15)	79.6	0.783	0.887	−9.8%
Mild Blur (σ = 1.0)	86.9	0.847	0.924	−2.5%
Severe Blur (σ = 3.0)	77.1	0.758	0.869	−12.3%
Combined (Moderate)	74.8	0.735	0.851	−14.6%

## Data Availability

The data presented in this study are available on request from the corresponding author due to privacy and ethical restrictions.

## Appendix A. Technical Implementation of Counterfactual Geometry Diffusion

### Appendix A.1. Geometric Index Manipulation in Latent Space

The Counterfactual Geometry Diffusion (CGD) module implements controlled geometric alterations through a multi-stage latent space manipulation process. Given an input radiograph x and target geometric modification Δg (e.g., slip ratio increase of 0.15), the process operates as follows:

Stage 1: Encoder-based Latent Extraction

The input image x is encoded into latent representation z_0_ = E (x) using a pre-trained VAE encoder, where z_0_ ∈ ℝ^H × W × c^ represents the compressed spatial-semantic encoding.

Stage 2: Geometry-Conditioned Latent Manipulation

A learned geometric transformation network G_θ_ maps target index changes to latent space modifications: z_1_ = G_θ_ (z_0_, Δg), where G_θ_ is trained to associate specific geometric parameters with corresponding latent space directions. The network architecture consists of:

- Geometric encoder: φ (Δg) → ℝᵈ mapping index changes to embedding space
- Spatial attention module: A (z_0_, φ(Δg)) identifying regions for modification
- Conditional transformation: z_1_ = z_0_ + λ · A (z_0_, φ(Δg)) · T (φ(Δg))

Stage 3: Diffusion-based Image Generation

The modified latent representation z_1_ serves as conditioning input for the reverse diffusion process: $\tilde{x}$ = D_ϕ_ (z_T_, z_1_), where D_ϕ_ represents the denoising network trained with geometry-aware conditioning.

### Appendix A.2. Training Procedure

The CGD module is trained using a combined loss function:

L_CGD_ = L_recon_ + λ_geom_ · L_geom_ + λ_consist_ · L_consist_

where

- L_recon_: reconstruction loss ensuring visual fidelity
- L_geom_: geometric consistency loss ensuring Δg is reflected in output indices
- L_consist_: semantic preservation loss maintaining non-target anatomical features

### Appendix A.3. Validation of Geometric Control

Generated images undergo automated verification where predicted indices are re-extracted using dRGM and compared against target modifications, ensuring geometric control precision within ±5% tolerance.

## Appendix B. Comprehensive Error Analysis

### Appendix B.1. Error Classification Framework

We conducted systematic analysis of the 171 misclassified cases (10.6% of total dataset) to identify failure patterns and inform future improvements. Errors were categorized into five primary types:

#### Appendix B.1.1. Subtle Pathology Errors (*n* = 73, 42.7%)

- Early Infection (*n* = 31): Cases with minimal disc space narrowing (<2 mm) without obvious endplate erosion, often misclassified as normal
- Mild Spondylolisthesis (*n* = 28): Grade I slips (slip ratio 0.15–0.25) misclassified due to positioning artifacts or measurement uncertainty
- Early SpA (*n* = 14): Minimal sacroiliac changes without clear syndesmophyte formation

#### Appendix B.1.2. Image Quality-Related Errors (*n* = 38, 22.2%)

- Poor contrast or motion blur affecting landmark detection
- Severe osteoporotic changes obscuring vertebral boundaries
- Overlapping structures in thoracic region complicating analysis

#### Appendix B.1.3. Anatomical Variant Errors (*n* = 26, 15.2%)

- Transitional lumbosacral vertebrae (*n* = 12)
- Congenital vertebral anomalies (*n* = 8)
- Post-surgical hardware artifacts (*n* = 6)

#### Appendix B.1.4. Multi-Pathology Cases (*n* = 22, 12.9%)

- Concurrent spondylolisthesis and infection (*n* = 13)
- SpA with superimposed degenerative changes (*n* = 9)

#### Appendix B.1.5. Labeling Ambiguity (*n* = 12, 7.0%)

- Cases where radiologist disagreement existed in original labeling
- Borderline cases with uncertain diagnostic classification

### Appendix B.2. Geometric Index Failures

Analysis of dRGM performance in error cases revealed:

- Slip ratio measurement failures in 34% of spondylolisthesis errors due to poor vertebral boundary detection
- Disc asymmetry calculation errors in 28% of infection misclassifications
- Cobb angle estimation problems in severe scoliotic cases affecting 15% of errors

### Appendix B.3. Clinical Implications

Error analysis reveals that model failures predominantly occur in clinically challenging cases where human inter-observer agreement is typically lower. This suggests the system performs comparably to human diagnostic uncertainty patterns, supporting its potential utility as a second-reader tool while highlighting the need for confidence scoring and uncertainty quantification in clinical deployment.

## Appendix C

**Algorithm A1:** Reproducible Training Pipeline for SpineNeuroSym
Input:
Raw DICOM spinal X-ray dataset D_raw
Config files:
dataset.yaml
krd_stage1.yaml
graph_stage2.yaml
end2end_stage3.yaml
Output:
Final trained model M*
Evaluation metrics (Accuracy, Macro-F1, AUROC, …)
1: # Environment setup
2: CreateCondaEnv(name = “spineneurosym”, python = 3.9)
3: InstallPyTorchWithCUDA()
4: InstallDependencies(requirements.txt)
5: # Dataset preparation
6: D_img ← ConvertDICOMtoPNG(D_raw, size = 512×512, mode = “16-bit grayscale”)
7: Split D_img into D_train, D_val, D_test with ratio 70/15/15 (stratified)
8: AnnotateSparseKeypoints(D_train, ratio = 0.15)
9: EnsureAnnotationQuality(kappa_target > 0.8)
10: # Build dataloaders using dataset.yaml
11: ApplyTrainTransforms(D_train) # rotation, flip, noise, normalize
12: CreateDataLoaders(D_train, D_val, D_test)
13: # ------------------------------
14: # Stage 1 – KRD Training
15: # ------------------------------
16: M_krd ← InitializeModel(config = “krd_stage1.yaml”)
17: for epoch = 1 to 50 do
18: for each batch B in D_train do
19: y_hat ← M_krd.forward(B)
20: L_krd ← ComputeKrdLoss(y_hat, B.labels)
21: Backpropagate(L_krd)
22: UpdateParameters(M_krd)
23: end for
24: ValidateOn(D_val) and update best checkpoint
25: end for
26: SaveBestCheckpoint(M_krd, path = “checkpoints/krd_best.ckpt”)
27: # ------------------------------
28: # Stage 2 – Graph Construction Training
29: # ------------------------------
30: M_graph ← InitializeModel(config = “graph_stage2.yaml”)
31: LoadCheckpoint(M_graph, “checkpoints/krd_best.ckpt”)
32: for epoch = 1 to 50 do
33: for each batch B in D_train do
34: y_hat ← M_graph.forward(B)
35: L_graph ← ComputeGraphLoss(y_hat, B.labels)
36: Backpropagate(L_graph)
37: UpdateParameters(M_graph)
38: end for
39: ValidateOn(D_val) and update best checkpoint
40: end for
41: SaveBestCheckpoint(M_graph, path = “checkpoints/graph_best.ckpt”)
42: # ------------------------------
43: # Stage 3 – End-to-End Optimization
44: # ------------------------------
45: M_e2e ← InitializeModel(config = “end2end_stage3.yaml”)
46: LoadCheckpoint(M_e2e, “checkpoints/graph_best.ckpt”)
47: for epoch = 1 to 50 do
48: for each batch B in D_train do
49: y_hat ← M_e2e.forward(B)
50: L_krd ← ComputeKrdLoss(y_hat, B.labels)
51: L_graph ← ComputeGraphLoss(y_hat, B.labels)
52: L_sym ← ComputeSymbolicConsistencyLoss(y_hat)
53: L_total ← WeightedSum(L_krd, L_graph, L_sym)
54: Backpropagate(L_total)
55: UpdateParameters(M_e2e)
56: end for
57: ValidateOn(D_val) and update best checkpoint
58: end for
59: SaveBestCheckpoint(M_e2e, path = “checkpoints/final_model.ckpt”)
60: M* ← LoadModel(“checkpoints/final_model.ckpt”)
61: # ------------------------------
62: # Evaluation on test set
63: # ------------------------------
64: SetRandomSeed(42)
65: predictions, labels ← EmptyLists()
66: for each batch B in D_test do
67: with NoGradient():
68: y_hat ← M*.forward(B)
69: Append(predictions, y_hat)
70: Append(labels, B.labels)
71: end for
72: accuracy ← ComputeAccuracy(predictions, labels)
73: macro_f1 ← ComputeMacroF1(predictions, labels)
74: auroc ← ComputeAUROC(predictions, labels)
75: Report(accuracy, macro_f1, auroc)
76: return M*, {accuracy, macro_f1, auroc}
End Algorithm

## Appendix D. Educational Tutorial for Clinical Implementation

### Appendix D.1. Understanding the SpineNeuroSym Pipeline

This tutorial provides step-by-step guidance for clinicians and researchers implementing SpineNeuroSym in clinical workflows.

#### Appendix D.1.1. Conceptual Overview

SpineNeuroSym processes spinal X-rays through four main stages:

1. Anatomy Discovery: Automatically locates vertebral centroids and disc spaces
2. Graph Construction: Organizes anatomical landmarks into structured representation
3. Geometric Analysis: Computes clinically relevant measurements (slip ratio, Cobb angles)
4. Diagnostic Classification: Combines geometric and imaging features for diagnosis

#### Appendix D.1.2. Clinical Interpretation Guide

Understanding Geometric Indices:

- Slip Ratio: Measures vertebral displacement (normal < 0.15, Grade I: 0.15–0.25, Grade II: 0.25–0.50)
- Disc Asymmetry: Quantifies left-right disc height differences (normal < 0.10, pathological > 0.20)
- SI Joint Spacing: Evaluates sacroiliac joint symmetry (normal difference < 2 mm)
- Cobb-like Angles: Approximates spinal curvature (cervical: 20–45°, thoracic: 25–50°, lumbar: 40–75°)

#### Appendix D.1.3. Integration with Clinical Workflow

Step 1: Image Acquisition Standards

- Standard AP/lateral projections with patient centered
- Optimal exposure parameters (80–120 kVp, 10–40 mAs)
- Minimal motion artifacts and appropriate collimation

Step 2: System Input Requirements

- DICOM format preferred, PNG acceptable
- Minimum resolution 1024 × 1024 pixels
- Proper patient positioning with visible anatomical landmarks

Step 3: Output Interpretation

- Review predicted diagnostic category with confidence score
- Cross-validate geometric indices against visual assessment
- Use system as “second reader” rather than definitive diagnosis

#### Appendix D.1.4. Quality Assurance Protocol

Monthly Calibration Checks:

1. Process standardized phantom images
2. Verify geometric index accuracy within ±5%
3. Monitor system performance metrics
4. Review discrepant cases with radiologist feedback

#### Appendix D.1.5. Troubleshooting Common Issues

Poor Landmark Detection:

- Check image quality and positioning
- Verify adequate vertebral visibility
- Consider manual landmark correction if available

Unexpected Index Values:

- Review patient anatomy for variants/anomalies
- Cross-check with manual measurements
- Consider clinical context (prior surgery, hardware)
